# Respiratory Viral Infections in Patients With Cancer or Undergoing Hematopoietic Cell Transplant

**DOI:** 10.3389/fmicb.2018.03097

**Published:** 2018-12-12

**Authors:** Diego R. Hijano, Gabriela Maron, Randall T. Hayden

**Affiliations:** ^1^Department of Infectious Diseases, St Jude Children's Research Hospital, Memphis, TN, United States; ^2^Department of Pathology, St Jude Children's Research Hospital, Memphis, TN, United States

**Keywords:** respiratory infection, virus, cancer, immunocompromised, hematopoietic cell transplant, RSV, influenza, child

## Abstract

Survival rates for pediatric cancer have steadily improved over time but it remains a significant cause of morbidity and mortality among children. Infections are a major complication of cancer and its treatment. Community acquired respiratory viral infections (CRV) in these patients increase morbidity, mortality and can lead to delay in chemotherapy. These are the result of infections with a heterogeneous group of viruses including RNA viruses, such as respiratory syncytial virus (RSV), influenza virus (IV), parainfluenza virus (PIV), metapneumovirus (HMPV), rhinovirus (RhV), and coronavirus (CoV). These infections maintain a similar seasonal pattern to those of immunocompetent patients. Clinical manifestations vary significantly depending on the type of virus and the type and degree of immunosuppression, ranging from asymptomatic or mild disease to rapidly progressive fatal pneumonia Infections in this population are characterized by a high rate of progression from upper to lower respiratory tract infection and prolonged viral shedding. Use of corticosteroids and immunosuppressive therapy are risk factors for severe disease. The clinical course is often difficult to predict, and clinical signs are unreliable. Accurate prognostic viral and immune markers, which have become part of the standard of care for systemic viral infections, are currently lacking; and management of CRV infections remains controversial. Defining effective prophylactic and therapeutic strategies is challenging, especially considering, the spectrum of immunocompromised patients, the variety of respiratory viruses, and the presence of other opportunistic infections and medical problems. Prevention remains one of the most important strategies against these viruses. Early diagnosis, supportive care and antivirals at an early stage, when available and indicated, have proven beneficial. However, with the exception of neuraminidase inhibitors for influenza infection, there are no accepted treatments. In high-risk patients, pre-emptive treatment with antivirals for upper respiratory tract infection (URTI) to decrease progression to LRTI is a common strategy. In the future, viral load and immune markers may prove beneficial in predicting severe disease, supporting decision making and monitor treatment in this population.

## Introduction

Respiratory viruses represent the most common infections in children (Tregoning and Schwarze, 2010; Meissner, 2016; Kou et al., 2018). In healthy children, they usually cause upper respiratory tract infections (URTI) with a benign, self-limited course (Tregoning and Schwarze, 2010; Meissner, 2016). However, immunocompromised hosts, particularly those undergoing transplant are particularly vulnerable to severe infections (Whimbey et al., 1996, 1997; Couch et al., 1997; Englund et al., 1999, 2011; Englund, 2001; Kim et al., 2007; Campbell et al., 2015). Furthermore, the spectrum of immunocompromised patients has expanded over the past decade owing to prolonged survival of those with various malignancies and to advances in hematopoietic cell transplantation (HCT) (Englund, 2001; Englund et al., 2011; Fisher et al., 2017). As diagnostic test improve and virus are detected more rapidly, the burden of these infections and their clinical outcomes have been progressively recognized (Englund et al., 1996, 1999; Englund, 2001). Although respiratory infections can be due to opportunistic infections, uncommon or atypical organisms, community respiratory virus are usually the cause of hospitalization in these patients (Englund et al., 1999, 2011; Fisher et al., 2017).

Respiratory viral infections include RNA viruses such as paramyxoviruses (RSV, parainfluenza, HMPV), orthomyxoviruses (influenza), picornaviruses (rhinovirus), and coronaviruses, as well as DNA viruses such as adenovirus, bocavirus, and polyomaviruses (Whimbey et al., 1996, 1997; Englund et al., 1999; Kim et al., 2007). These RNA viruses are among the most common causes of respiratory infection in immunocompromised patients (Whimbey et al., 1997; Hirsch et al., 2013) (Table 1). Although the annual activity of these viruses is variable, distinct patterns have been described. They cause seasonal outbreaks, and most are predominantly seen during the winter (Whimbey et al., 1996, 1997; Kim et al., 2007).

**Table 1:** Taxonomy and virologic properties of the major human respiratory RNA viruses^a^.

**Virus**	**Family**	**Size (nm)**	**RNA genome**	**Envelope**	**Genetic or antigenic types**
Respiratory syncytial virus	*Paramyxoviridae*	120–200	Linear ss(–)	Yes	Antigenic subgroups A and B with 10 A genotypes and 13 B genotypes
Influenza virus	*Orthomyxoviridae*	80–120	Segmented ss(–)	Yes	3 antigenic types (A, B, C); A has 3 HA and 2 NA human subtypes
Parainfluenza virus	*Paramyxoviridae*	120–180	Linear ss(–)	Yes	4 serotypes (1, 2, 3, 4); subtypes 4a and 4b
Metapneumovirus	*Paramyxoviridae*	120–180	Linear ss(–)	Yes	Subtypes A and B; subgroups A1/A2 and B1/B2, respectively
Rhinoviruses	*Picornaviridae*	20–27	Linear ss(+)	No	>100 antigenic types
Coronaviruses	*Coronaviridae*	80–160	Linear ss(+)	Yes	6 genotypes (229E, OC43, NL63, HKU1, SARS-CoV, MERS-CoV)

a *ss(–), single-stranded negative-sense RNA; ss(+), single-stranded positive-sense RNA; HA, hemagglutinin; NA, neuraminidase; SARS-CoV, severe acute respiratory syndrome-coronavirus; MERS-CoV, Middle East respiratory syndrome-coronavirus. Reproduced from Hodinka (2016)*.

Clinical presentation can range from asymptomatic viral shedding to severe respiratory distress (Hakim et al., 2016b; Fisher et al., 2017). Severity of disease depends on intrinsic viral factors, as well on patient characteristics including underlying diagnosis and treatment, with myelosuppression and HCT bearing the highest morbidity and mortality (Kim et al., 2007; Hakim et al., 2016b; Fisher et al., 2017). Adverse outcomes are far more likely in immunocompromised patients than in non-immunocompromised individuals and include progression to pneumonia, respiratory failure, and increased mortality rates (Kim et al., 2014). In immunocompromised adults with cancer, progression to LRTI ranges from 30 to 50%, and for those who develop pneumonia, mortality can be as high as 75% (Hirsch et al., 2013; Renaud et al., 2013; Chemaly et al., 2014; Campbell et al., 2015; Hutspardol et al., 2015; Chu et al., 2016). Mortality associated with respiratory viruses in children undergoing HCT varies, usually ranging between 10 and 14% but can be as high as 30% (Ljungman et al., 2001; Luján-Zilbermann et al., 2001; Hutspardol et al., 2015). Although significantly lower than in adults, mortality remains considerably higher than that for the general population. Long term complications associated with respiratory viral infections, such as airflow obstruction and bronchiolitis obliterans, have developed in HCT and lung transplant recipients (Campbell et al., 2015; Chu et al., 2016).

A focus of interest has been to determine and assess risk factors for progression to LRTI with the goal to detect patients who might benefit from interventions that could prevent clinical progression. Age, lymphopenia, high-dose total body irradiation and presence of co-pathogens are significant risk factors for progression to LRTI (El Saleeby et al., 2008; Waghmare et al., 2013; Kim et al., 2014). Clinical scores have been developed for adults undergoing HCT and in healthy children with RSV infection to better predict severity (Shah et al., 2014; Caserta et al., 2017). However, the former has not been validated in children and the latter lacks important features surrounding this particular sub group of patients.

## Diagnostic

Diagnostic testing for respiratory viruses has evolved tremendously in the past several years. This is equally true for HCT patients as it has been for immunocompetent patients, where the same testing modalities are utilized. Molecular amplification techniques have largely supplanted cell culture as the primary means of detecting and identifying viruses in clinical diagnostic samples (Mahony, 2008; Mahony et al., 2011). Antigen-based detection is used in some acute care settings, but typically shows inferior sensitivity and specificity compared to molecular methods. PCR is the most common amplification technique, with FDA cleared or approved *in vitro* diagnostic tests available commercially to detect all common respiratory viral pathogens (Caliendo, 2011) (Table 2). These are marketed as single analyte assays (such as tests that detect only influenza A), as assays detecting only a small number of analytes (for example, detecting only influenza and RSV), and as broad-panel test (detecting 12–20 different pathogens) (Rand et al., 2011; Hammond et al., 2012; Hayden et al., 2012; Popowitch et al., 2013; Salez et al., 2015). Often the latter also include some common bacterial agents of infection. Such molecular tests generally have a high degree of sensitivity and specificity, with a much more rapid time to result compared to culture. Larger, multiplexed panels can be advantageous in an immunocompromised population as even the detection of pathogens without available specific antiviral therapies can have important implications for infection control and potentially for decisions surrounding the time of transplant. Furthermore, symptoms can be atypical, making it difficult to predict the most likely agents of infection with any degree of clinical certainty. Such highly sensitive tests raise numerous questions, however. Despite uncertain clinical significance, the incidence of detectable multi-viral infections tends to be increased using these methods, sometimes with four or more agents detected simultaneously. The advent of molecular testing has also increased the time of detectable viral shedding which is often lengthened in immunocompromised patients, and the risk of spread or recurrence during periods of asymptomatic shedding is unknown. Quantitative methods (as noted elsewhere in this work) may shed light, both on viral dynamics and on the clinical implications of asymptomatic shedding. Such information awaits increased availability of these tests, together with publication of more studies in this high-risk population.

**Table 2:** Laboratory methods for diagnosis of the major human respiratory RNA viruses^a^.

**Methods**	**Sensitivity**	**Specificity**	**Test time**	**Clinical usefulness**
**VIRUS CULTURE SYSTEMS**				
Conventional tube	Low-moderate	High	3–14 d	Capable of growing any virus; involves considerable time, labor, and resources
Shell vial or microwell plate	Low-moderate	High	1–3 d	Rapid centrifugation-assisted cultures for select viruses; less sensitive than tube cultures
**DIRECT ANTIGEN DETECTION TESTS**				
Immunofluorescence	Moderate	Moderate-high	1–2 h	Rapid, multianalyte detection; moderately complex and subjective reading
Immunoassays	Low-moderate	Moderate-high	≤ 15 min	Fast and simple to use; amenable to point-of-care testing
Nucleic acid amplification tests	High	High	15 min−8 h	New reference standard; superior performance characteristics
Serology	NA	NA	NA	Not useful for diagnosis; used for research and epidemiologic studies

a *d, days; h, hours; min, minutes. Reproduced from Hodinka (2016)*.

Several authors have studied the yield and correlation of respiratory samples from upper airway and lower airway in the setting of LRTI. Hakki et al. analyzed 72 paired nasopharyngeal (NP) and bronchoalveolar lavage fluid specimens from immunocompromised adult patients for respiratory viruses. NP samples had a positive and negative predictive value of 86.4 and 94% respectively. In three sets the NP sample was negative and the BAL was positive (Hakki et al., 2014). Azadeh et al. report that 20% of patients with an initial negative NP wash had a positive BAL sample, suggesting that a BAL may be valuable when there are concerns for LRTI and the NP wash is negative (Azadeh et al., 2015). This has been particularly reported by several groups in the setting of severe pandemic H1N1 influenza infection (Mulrennan et al., 2010; Yeh et al., 2010; Lee et al., 2011).

Susceptibility testing is limited to influenza. Emergence of universal resistance of IV A H1N1 and IV A H3N2 to adamantanes, as well as rapid emergence of oseltamivir resistant IV A H1N1 led to increased efforts to closely monitor drug resistance (Renaud et al., 2010, 2011a,b; Okomo-Adhiambo et al., 2014, 2015). Both phenotypic and genotypic assays have developed, but genotypic methods are most commonly used for routine testing to detect neuraminidase inhibitor resistance of influenza viruses in clinical specimens (Hirsch et al., 2013; Marjuki et al., 2013; Okomo-Adhiambo et al., 2013; Laplante and St George, 2014; Tamura et al., 2015).

## Isolation Control Practices

Several strategies have been suggested to decrease the risk of hospital transmission of respiratory viruses. Hand hygiene, screening visitors for respiratory symptoms, education of healthcare personal to avoid caring for patients if they are sick and isolation of patients with respiratory symptoms are among the most widely strategies implemented across institutions (Dykewicz, 2001; Tomblyn et al., 2009; Engelhard et al., 2013; Hirsch et al., 2013). Molecular testing has allowed for more rapid detection of viral etiology becoming an important tool to guide prevention practices. Whole genome sequencing—next generation sequencing is emerging as a potent tool to further enhance outbreak investigation and has been successfully applied in rhinovirus, influenza, and RSV (Gilchrist et al., 2015; Greninger et al., 2017; Kothari et al., 2017; Zhu Y. et al., 2017; Houlihan et al., 2018). A particular challenge in this population is the high proportion of asymptomatic patients with respiratory viral infections and whether isolation in the absence of symptoms is indicated (Raad et al., 1997; Waghmare et al., 2016). Quantitative methods may aid useful information on determining the infectious risk in these patients.

Patients with respiratory symptoms should be put immediately on contact and droplet isolation. At St Jude we use clinical symptoms and viral etiology to determine isolation strategies. We stop isolation in patients with infections due to rhinovirus, enterovirus, coronavirus, or unidentified etiology after 48 h of resolution of symptoms with no retesting needed. Patients with respiratory infection due to RSV, influenza, HMPV, or HPIV require both resolution of symptoms and a negative test to be taken off isolation.

## Treatment

Although advances in the treatment of respiratory infections have been made, management of viral infections in this population remains a challenge (Ljungman et al., 2001; Casper et al., 2010; Chemaly et al., 2014; Waghmare et al., 2016; Shahani et al., 2017). Treatment is primarily supportive and includes, fluid replacement, oxygen for hypoxia and mechanical ventilation if necessary (Meissner, 2016; Kou et al., 2018). Bronchodilators may provide some benefit (Meissner, 2016; Kou et al., 2018). Given the significant mortality rate in immunocompromised host, antiviral therapy should be considered when available (Casper et al., 2010; Renaud and Englund, 2012; Hirsch et al., 2013; Waghmare et al., 2016; Shahani et al., 2017). However, antiviral options are scarce and limited to influenza and, to a lesser extent, RSV (Table 3) (Casper et al., 2010; Chemaly et al., 2014; Waghmare et al., 2016; Jorquera and Tripp, 2017; Shahani et al., 2017). In addition, adjunctive therapy such as intravenous immunoglobulin (IVIG) or monoclonal antibodies, such as palivizumab, have shown mixed results (Ghosh et al., 2000; de Fontbrune et al., 2007; Hirsch et al., 2013; Falsey et al., 2017). Several antivirals and monoclonal antibodies are at different stages of development for the treatment of respiratory viruses (Table 4).

**Table 3:** Current approved antivirals for treatment of the major human respiratory RNA viruses.

**Virus**	**Antiviral**	**Drug class**	**Dose/Duration**		**Side effects**	**Resistance**
Respiratory syncytial virus	Ribavirin	Nucleoside analog	Children/Adults: Inhaled Oral	2 grams over 2 h TID Loading dose of 600 mg 200 mg TID on day 1 400 mg TID on day 2 Then maximum 30/mg/kg/day	Bronchospasm Hemolysis, liver and renal toxicity	None reported
Influenza virus	Oseltamivir	NAI	Adults Children >40 kg ≥ 12 24–40 kg months 16–23 kg 15 kg or less	75 mg PO twice per day 75 mg PO twice per day 60 mg PO twice per day 45 mg PO twice per day 30 mg PO twice per day	Nausea, vomiting	Influenza A(H1N1) virus strains H275Y substitution leads to resistance
			Children < 12 months	3 mg/kg/dose PO twice per day		
			Longer duration (10 days) for immunocompromised individuals			
	Zanamivir	NAI	Adults	Two 5-mg inhalations (10 mg total) twice per day	Bronchospasm, diarrhea, nausea, headache, dizziness	Influenza A (H1N1) with both an H275Y and E119D or E119G. NA substitution lead to resistance
			Children (age, 7 years or older)	Two 5-mg inhalations (10 mg total) twice per day		
	Peramivir	NAI	Adults	600 mg single dose (IV)	Neutropenia, diarrhea	Influenza A(H1N1) virus strains with H275Y substitution leads to resistance
			Children (age, 29 days of life or older)	N/A		
			Longer duration (5 days) for immunocompromised individuals			
	Amantadine^*^	M2 inhibitors	≥10 years and ≥40 kg ≥10 years and <40 kg 1–9 years.	100 mg PO twice daily 5 mg/kg/day PO in 2 doses 5 mg/kg/day PO in 2 doses	Cardiac, neurologic and gastrointestinal events; neutropenia	High prevalence of resistance in all Influenza A (H3N2) and (H1N1) pdm09 Not active against Influenza B
	Rimantadine^*^	M2 inhibitors	≥10 years/Adolescents 1 − 9 years	5 mg/kg/day PO in 2 doses 6.6 mg/kg/day PO in 2 doses	Neurological and cardiac events	Same as for Amantadine
	Baloxavir marboxil	Endonuclease inhibitor	≥ 12 years and ≥80 kg ≥12 years and <80 kg.	80 mg PO once 40 mg PO once	Diarrhea, bronchitis	Influenza A (H3N2) and (H1N1) with substitutions I38F/M/F show reduced susceptibility.
Parainfluenza virus	None licensed					
Metapneumovirus	None licensed					
Rhinovirus	None licensed					
Coronavirus	None licensed					

* *Not recommended given high prevalence of resistance for influenza A strains. Adapted from Englund et al. (1990, 1994), Knight et al. (1991), Boeckh et al. (2007), Casper et al. (2010), Renaud et al. (2010, 2011a,c), Hirsch et al. (2013), Waghmare et al. (2016), Shahani et al. (2017), AAP (2018), and Hayden et al. (2018)*.

**Table 4:** Antivirals and monoclonal antibodies on the pipeline for treatment of the major human respiratory RNA viruses.

**Virus**	**Antiviral**	**Drug class/mechanism of action**	**Route of administration**	**Company**
Respiratory syncytial virus	ALS-8176 (a.k.a Lumicitabine)	Nucleoside analog	Orally	Alios BioPharma
		RNA-dependent RNA-polymerase (RdRp)		
	GS-5806 (a.k.a Presatovir)	Fusion inhibitor^*^	Orally	Gilead
	VP-14637 (aka MDT-637)	Fusion inhibitor^*^	Inhaled	ViroPharma
	JNJ-53718678	Fusion inhibitor	Orally	Johnson & Johnson
	BTA-C585 (a.k.a Enzaplatovir)	Fusion inhibitor^*^	Orally	Vaxart
	AK-0529	Fusion inhibitor	Orally	Ark Biosciences
	RSV604	Nucleoprotein inhibitor	Orally	Astra Zeneca
	ALN-RSV01	Nucleoprotein inhibitor	Orally	AlnylamPharmaceuticals
	Palivizumab (Synagis)	Monoclonal antibody	Intramuscular	MedImmune
	REGN2222 (a.k.a Suptavumab)	Monoclonal antibody	Intramuscular	RegeneronPharmaceuticals
	MEDI8897	Monoclonal antibody	Intramuscular	MedImmune
	ALX-0171	Monoclonal antibody	Inhaled	Ablynx
Influenza virus	DAS181 (a.k.a.Fludase)	Targets the Viral Receptor (Sialic Acid)	Inhaled	Ansun BioPharma
	Laninamivir (CS-8958)	Long-acting neuraminidase inhibitors	Inhaled	Biota
	Favipiravir (T705)	Nucleotide analogRNA polymerase	Orally	Toyama Chemical
	JNJ-63623872 (a.k.a VX-787)	Nonnucleoside inhibitor	Orally	Janssen
	Nitazoxanide (a.k.a NT-300)	ThiazolidesInhibits the maturation of influenza virus HA	Orally	Romark Laboratories
	MEDI8852	Monoclonal antibody	Intravenous	AstraZeneca
	VIS410	Monoclonal antibody	Intravenous	Visterra
Parainfluenza virus	DAS181 (a.k.a.Paradase)	Targets the Viral Receptor (Sialic Acid)	Inhaled	Ansun BioPharma
Human Metapneumovirus	MAb 338	Monoclonal antibody	Intravenous	Medimmune
Human Rhinovirus	Vapendavir	Binds to the RhV VP1 capsid	Orally	Aviragen Therapeutics

* *inhibit RSV fusion through a similar mechanism, and RSV variants exhibiting drug resistance have displayed cross-resistance to these inhibitors. Adapted from (Douglas et al. (2005); Triana-Baltzer et al. (2009b); Boeckh and Englund (2010); Kiso et al. (2010); Sleeman et al. (2010); Watanabe et al. (2010); Guzmán-Suarez et al. (2012); Moss et al. (2012); Adedeji et al. (2013); Matz (2013); Clark et al. (2014); DeVincenzo et al. (2014, 2015); Rossignol (2014); Byrn et al. (2015); Battles et al. (2016); Waghmare et al. (2016); Coates et al. (2017); Heylen et al. (2017); Jorquera and Tripp (2017); Kim et al. (2017); Koszalka et al. (2017); Mejias et al. (2017); Roymans et al. (2017); Shahani et al. (2017); Heo (2018); Omoto et al. (2018); Rossey et al. (2018), and Stevens et al. (2018)*.

## RSV

RSV is a single stranded, negative-sense RNA virus within the family *Paramyxoviridae*. Its genome transcribes to produce 11 proteins: NS1, NS2, N, P, M, SH, G, F, M2-1, M2-2, and L (Papenburg and Boivin, 2010; Tregoning and Schwarze, 2010). The attachment glycoprotein (G) and the fusion (F) glycoprotein are the two major glycoproteins on the surface of RSV. They control the initial phases of infection (McLellan et al., 2013). F protein is the main target for antiviral development. However, both G and F glycoproteins are targeted by neutralizing antibodies during natural infection (Capella et al., 2017; Melero et al., 2017; Tian et al., 2017). There is a single antigenic type of RSV, divided into two subgroups, A and B. Usually one of these groups predominates on an epidemic. The significant of the variants and its role on disease severity is not well-understood (Hall et al., 1990; Walsh et al., 1997, 2018; Devincenzo, 2004).

Respiratory syncytial virus (RSV) is the most common cause of lower respiratory tract infection in children. Premature infants, infants with cardiac disease, and severely immuno-compromised patients experience increased morbidity and mortality (Domachowske and Rosenberg, 1999; DeVincenzo et al., 2003; Devincenzo, 2004; Hall et al., 2009, 2013). RSV infections resembles the same seasonal pattern in patients with HCT and/or other hematological diseases and its occurrence is a result of increased risk of infection from the community, household contacts, and nosocomial transmission (Hall, 2001; Hall et al., 2009, 2013). In a single center study, Hakim and colleagues determined the epidemiology of respiratory viruses on children with acute lymphoblastic leukemia. They reported that RSV was the second most common respiratory accounting for 31% of all episodes of respiratory viral infections (Hakim et al., 2016b). Hirsch et al. described that RSV infection in pediatric patients with acute myeloid leukemia can be up to 2.2% (Hirsch et al., 2013). Fisher et al found that RSV was the third most common respiratory viral infection in pediatric patients undergoing HCT after RhV and PIV, with an all cause fatality rate of 10% (Fisher et al., 2017).

RSV LRTI is associated with increased rate of hospitalization and mortality. Several risk factors have been described for RSV LRTI (Seo et al., 2013; Chemaly et al., 2014; Kim et al., 2014). El-Saleeby and colleagues found that younger age and lymphopenia were significantly associated with RSV LRTI in children (El Saleeby et al., 2008). In Hakim's study a lower nadir ALC also associated with RSV LTRI (Hakim et al., 2016b). In fact, RSV, as compared to other virus, was more likely to be associated with LRTI, accounting for 58% of all viral episodes of LRTI (Hakim et al., 2016b). Age, neutropenia, lymphocytopenia, graft-vs.-host disease, use of myeloablative conditioning regimens, use of corticosteroids, a recent HCT, and pre-engraftment are the most significant risk factors included in an index developed by investigators at University of Texas MD Anderson Cancer Center to categorize patients into prognostic risk groups (Shah et al., 2014). Waghmare et al. explored several risk factors for RSV mortality in patients undergoing HCT with RSV infection, including the presence of RSV viremia. This was present in one third of the patients after development of LRTI. While neutropenia and mechanical ventilation were significantly associated with RSV viremia, no effect from lymphopenia or steroids use was observed. In this cohort viremia was independently associated with mortality. Treatment with aerosolized ribavirin was associated with improved outcomes, including decrease overall mortality and mortality due to respiratory failure (Waghmare et al., 2013).

Attempts to correlate viral load with RSV disease severity have yielded mixed results (Devincenzo, 2004; DeVincenzo et al., 2005; Gerna et al., 2008; Franz et al., 2010; El Saleeby et al., 2011; Walsh et al., 2018). On one hand, DeVincenzo et al. noted that patients that were intubated had higher viral load than those who were not, and that higher viral load was associated with longer stayed in the hospital (Buckingham et al., 2000; DeVincenzo and Buckingham, 2002; DeVincenzo et al., 2005). However, other studies have not found such association (Gerna et al., 2008; Franz et al., 2010; Lukens et al., 2010). Fuller et al. reported an association of viral load and disease severity in patients under 5 years of age but not in older patients (Fuller et al., 2013). The inconsistency of results may be due to technical difficulties of precisely quantifying virus load at a mucosal surface, or perhaps optimal analysis may require sampling over several days. At St. Jude, we have developed a droplet digital PCR assay to quantify RSA A and RSV B in respiratory specimens. Initial clinical validation was performed with 73 samples from 19 patients with RSV infection. RSV viral load was significantly higher in febrile patients <5 years old, whereas no statistical difference was noted with older patients. Patients who presented with either cough or nasal congestion had significantly higher viral load when compared to those who did not. Patients presenting with all 3 signs and symptoms had higher viral load when compared to those that only presented with one symptom. Eight patients with RSV infection were treated with Ribavirin. Seven of them received inhaled ribavirin and one received IV ribavirin since the patient was on mechanical ventilation. Viral load upon completion of treatment was significantly reduced (Data not published). Viral load testing for RSV is not widely available for clinical diagnostic use, and no FDA-approved methodology is currently on the market.

Ribavirin is a nucleoside analog that resembles guanosine and acts by inhibiting the dehydrogenase enzyme impairing the synthesis of guanosine triphosphate leading to reduce cellular deposits of guanidine necessary for viral growth (Shahani et al., 2017). Initiation and elongation of RNA is hampered impeding viral protein synthesis (Knight et al., 1991; Chemaly et al., 2014, 2016). Aerosolized ribavirin is the only FDA-approved treatment of severe RSV-LRTIs in hospitalized infants and young children with underlying compromising conditions (prematurity, cardiopulmonary disease, or immunosuppression) (Chemaly et al., 2014, 2016; Waghmare et al., 2016). Furthermore, ribavirin-based antiviral therapy is recommended by European guidelines for leukemia patients and HCT recipients at high risk of complications (Hirsch et al., 2013).

Shah et al. performed a systematic review based mainly on retrospective studies and found that ribavirin-based therapy was associated with a significant decrease in progression from URTIs to LRTIs. Furthermore, ribavirin alone or in combination with immunomodulatory therapy improved mortality rates when compared to no therapy in adult HCT recipients (Shah and Chemaly, 2011). This topic has recently gained a lot of interest given the increase in price of inhaled ribavirin from $6105 per day in January 2013 to $29,953 per day, based on average whole- sale price (Chemaly et al., 2016). As a consequence of this rise, a 5-day and a 10-day treatment course would cost $149,765 and $299,530 respectively. However, the oral formulation is ~$25 per day depending on dosing strategy (Chemaly et al., 2016; Waghmare et al., 2016). Some authors suggest that the oral formulation is not effective given low achievable plasma concentrations (Waghmare et al., 2016). Other studies show benefit of using oral ribavirin for adult patients with RSV infection (Damlaj et al., 2015; Foolad et al., 2017, 2018; Pasikhova et al., 2018). These unanswered questions are important given the difference in cost between the two formulations (Shah et al., 2016a).

Adjuvant therapy for RSV infection has also been controversial. Various other therapies such as intravenous immunoglobulin (IVIG), RSV hyperimmunoglobulin, and palivizumab, have been used for treatment (Shah and Chemaly, 2011; Shah et al., 2012; Waghmare et al., 2016; Shahani et al., 2017). Animal models and initial studies showed benefits of ribavirin plus RSV hyperimmunoglobulin compared to ribavirin alone (Gruber et al., 1987; Whimbey et al., 1995; Ottolini et al., 1999; Ghosh et al., 2000; Falsey et al., 2017). However, production of RSV IVIG has since been discontinued. In adult HCT recipients with RSV pneumonia, uncontrolled studies suggested that use of combination therapy with ribavirin and IVIG improved survival (Whimbey et al., 1995; Ghosh et al., 2000). However, combined use of ribavirin and IVIG has not been supported by a randomized trial. In forty allogenic HCT recipients with RSV infection, palivizumab failed to reduce progression to LRTI or mortality (de Fontbrune et al., 2007). Given the questionable efficacy and high cost of palivizumab (Ambrose and McLaurin, 2015), routine use has been discouraged; and practices vary widely among institutions.

**Figure 1:**
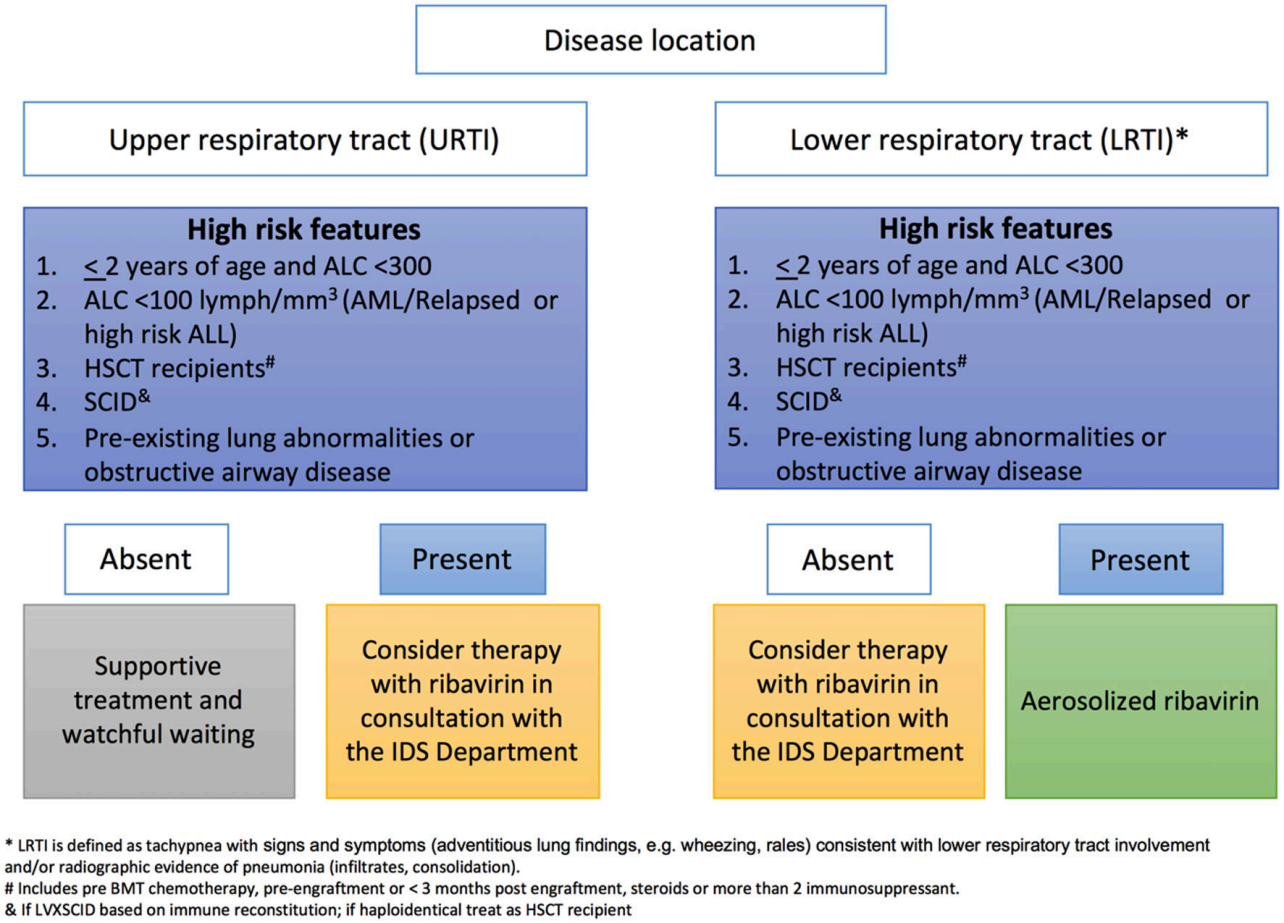
Institutional decision tree used at St Jude for inhaled Ribavirin therapy based on risk factors of patients infected with RSV.

At our institution, ribavirin is recommended for treatment of RSV URTI or LRTI in patients <2 years old with ALC below 300, or in patients of any age with an ALC below 100 who have a diagnosis of AML or relapsed or high risk ALL, those undergoing HCT or have pre-existing severe heart or lung disease (Figure 1). We do not use palivizumab as part of treatment, and only consider IVIG if total Ig is below 400 mg/dl. Other antivirals against RSV are at different stages of development (Table 4) (Jorquera and Tripp, 2017).

Palivizumab has been proven effective in reducing severe RSV infection in premature infants and children with chronic lung disease (AAP, 2014a,b). Although efficacy of palivizumab has not been assess in randomized trials in immunocompromised, prudent use of palivizumab in these population may be indicated. Kassis et al. described successfully control of a nosocomial outbreak of RSV in a bone marrow transplant unit where use of palivizumab reduced transmission of RSV in high risk group patients (Kassis et al., 2010).

At St. Jude, Palivizumab is administered in 5 monthly doses (November-March) to all patients with AML, SCID, or HCT recipients (including pre-HCT conditioning) who are ≤ 24 months of age and patients ≤ 12 months of age with a diagnosis of acute lymphoblastic leukemia. Other patients ≤ 24 months of age who are receiving intensive, immunosuppressive chemotherapy during the RSV season are considered for prophylaxis on a case by case basis.

## Influenza

Human influenza viruses (IVs) are members of the family *Orthomyxoviridae* and belong to three genera that contain single antigenically distinct species of the same name: influenza A, influenza B, and influenza C. They have a segmented, single stranded, negative sense RNA genome (Nicholson et al., 2003). The three IVs types are distinguished by antigenic differences in their nucleoprotein and matrix protein. The two main surface glycoproteins, hemagglutinin (HA) and neuraminidase (NA) are critical in the replication cycle and pathogenesis of IVS (Nicholson et al., 2003).

The seasonal prevalence of influenza infections in immunocompromised patients, closely parallels the community wide prevalence (Appiah et al., 2015; Bednarska et al., 2015). The incidence of influenza in transplant recipients is low. However, influenza infection remains a significant cause of morbidity and mortality in these patients (Weigt et al., 2011). The incidence in HCT recipients ranges from 1 to 3% (Nichols et al., 2004). Hakim et al. found that influenza was the most common virus pediatric patients with acute lymphoblastic leukemia (ALL), accounting for 38% of respiratory infections. In this cohort, the majority of patients presented with URTI, and only 6 had evidence of progression to LRTI. Of those, 3 had 2009 pandemic H1N1 influenza A virus, and one had RSV and influenza B virus co-infection, while the remaining 2 patients had influenza A virus that was not subtyped (Hakim et al., 2016b).

The clinical presentation of influenza does not differ considerably from that described in the general population. URTI symptoms consist of rhinitis and cough. Fever is usually seen in patients with pneumonia (Chemaly et al., 2006; Kumar et al., 2018). Less frequently, patients can present with gastrointestinal and neurological symptoms (Ison and Hayden, 2002; Peck et al., 2007). Risk factors associated with severity of infection and progression to LRTI have been described, including lymphopenia, steroids use, and delayed antiviral therapy (Ljungman et al., 2001, 2011; Martino et al., 2005; Chemaly et al., 2006; Khanna et al., 2009; Choi et al., 2011). Initiation of antiviral treatment within 48 h of symptoms has been associated with improved outcomes (Nichols et al., 2004; Choi et al., 2011; Espinosa-Aguilar et al., 2011). Tantawi et al. reported 90 children and adolescents with different cancer types who presented with LRTIs necessitating hospital admission. Respiratory viruses were identified in 37.7% of the patients, the majority with hematological malignancies. Influenza were the most common virus associated with hospital admission (influenza A 47%, influenza B 12%). All patients admitted to the ICU had bacterial co-infection. Those who received oseltamivir had a less severe clinical picture (Tantawy et al., 2015).

Respiratory complications, such as mechanical ventilation are associated with mortality in immunocompromised patients with influenza infection. The latter ranges from 6 to 28% and can be related to direct viral pathogenesis or to superimposed bacterial infections (Ljungman et al., 2001; Nichols et al., 2004; Choi et al., 2011; Renaud and Campbell, 2011; Fisher et al., 2017).

In pediatric cancer patients, antiviral treatment should be initiated as early as possible, even if patients have been symptomatic for over 48 h, since there may be benefits even with delayed therapy (AAP, 2018). While Influenza B is inherently resistant to M2 inhibitors (amantadine and rimantadine), influenza A has become resistant overtime. Hence, their use is limited. Neuraminidase inhibitors (NAIs), such as oseltamivir, zanamivir, and peramivir are now first line for prophylaxis and treatment of influenza A and B (Casper et al., 2010; Fiore et al., 2011; Chemaly et al., 2014). Oseltamivir is given by mouth, and its main side effects are nausea, vomiting and diarrhea. Inhaled zanamivir can cause bronchospasm, so it should be used cautiously in in patients with chronic lung disease. Intravenous zanamivir is available for compassionate use only. Peramivir is the only NAI approved for IV use (Ison and Hayden, 2002; Louie et al., 2012a,b; Ison, 2014). Studies comparing oseltamivir to peramivir have shown similar outcomes. However, the efficacy and safety of peramivir in immunocompromised patients needs further assessment (de Jong et al., 2014).

Most mutations conferring resistance to NAIs, such as H275Y in pandemic influenza A H1N1, do not lead to resistance in zanamivir, and its inhaled formulation has been successfully used to treat these patients (Renaud et al., 2011a; Fraaij et al., 2015; Waghmare et al., 2016). This is especially important, since immunocompromised patients are at increased risk of developing resistant mutants (Couturier et al., 2010; Renaud et al., 2010, 2011a,c; Carr et al., 2011b; Hamada et al., 2012; Fraaij et al., 2015). Combination therapy has poorly been studied and has not shown benefits. Some experts have recommended increased dosing of oseltamivir due to concerns of poor absorption in patients with mucositis or graft vs. host disease. However, the benefits of such intervention have not been conclusively demonstrated (Casper et al., 2010; Watcharananan et al., 2010). Length of therapy remains controversial. Given the median time to progression to LRTI and the potential for recurrence, longer treatment courses (10 days) have been suggested in this population (Khanna et al., 2009; Waghmare et al., 2016).

Endonuclease inhibitors are a new class of antiviral drugs (Clark et al., 2014; Byrn et al., 2015). Phase III development of Baloxavir marboxil (Xofluza, Shionogi & Co Ltd) is underway in the USA, EU and other countries (Heo, 2018; Omoto et al., 2018). Xofluza was approved in Japan on February 2018 and has recently been approved in the USA as single oral dose for the treatment of influenza A or B virus infection of patients 12 years of age and older (FDA, 2018). The main side effects described have been diarrhea and bronchitis. Amino acid substitutions to the endonuclease active site (I38F/T) have shown reduce susceptibility for Influenza A (H3N2) and (H1N1) strains (Hayden et al., 2018). Further studies need to be done to determine whether a single dose would be appropriate for immunocompromised patients.

Early diagnosis and isolation precautions are critical in preventing transmission. Post-exposure antiviral chemoprophylaxis should be given to any immunocompromised patient who comes in close contact with proven or suspected cases during the infectious period of the case (Fiore et al., 2011). The infectious period for persons with influenza is estimated to range from 1 day before up to 7 days after development of symptoms (Nicholson et al., 2003). If a patient comes into contact with a case in an immunocompetent host whose illness started more than 7 days prior, then chemoprophylaxis is not necessary. However, infants and immunocompromised patients shed the virus and remain contagious for a longer period. Post-exposure chemoprophylaxis should last up to 7 days after the last known exposure to influenza.

All immunocompromised patients 6 months of age or older should be vaccinated as soon as possible with the seasonal influenza vaccine (Ljungman, 2012; Rubin et al., 2014). Although its immunogenicity in immunocompromised patients is variable, and vaccine response is lower than in the general population, some studies show that when vaccinated patients get influenza the severity of infection is less that that compared to unvaccinated patients (Engelhard et al., 1993; Machado et al., 2005; Issa et al., 2011; Beck et al., 2012). High-dose trivalent inactivated influenza vaccine elicited a more robust immune response in children with cancer when compared to standard-dose vaccine (Hakim et al., 2016a). At our institution 2 doses of influenza vaccination are recommended, administered at least 28 days apart. Vaccination of family members and health care providers is also an important measure to prevent influenza infection in these patients (Carr et al., 2011a; Hakim et al., 2016a; Sykes et al., 2017).

## Parainfluenza

Parainfluenza viruses (PIVs) belong to the family *Paramyxoviridae* and have a structure similar to that of RSV with a negative sense, single-stranded RNA genome that encodes six common viral proteins. Hemagglutinin-neuraminidase (HN) and F glycoprotein promote viral attachment and fusion of the viral and host cell membranes (Hall, 2001; Henrickson, 2003). At present, 5 serotypes of Human PIVs (HPIVs) have been recognized: HPIV-1, HPIV-2, HPIV-3, HPIV-4a, and HPIV-4b. They differ in their frequency of occurrence, spectrum of clinical disease and epidemic patterns (Henrickson, 2003). PIV3 is the most common serotype producing infections. Infections can occur at any time during the year. However, during spring and summer there is a peak of activity (Hall, 2001; Henrickson, 2003).

A recent multicenter study aiming to define the epidemiology and outcomes of respiratory virus infection in pediatric HCT recipients showed PIV as the second most common virus. In this cohort RVI was associated with an all-cause case fatality rate of 11 and 8% of patients with HPIV infection died (Fisher et al., 2017). A large retrospective study of 1,554 children diagnosed with cancer found that PIV was the most common respiratory virus with an incidence of 54% (74/137). Median age was 3.9 years and 30% of the patients were HCT recipients (Maeng et al., 2012). Most of the patients presented with URTI and 20% of them progressed to LRTI with a median of 4 days from onset of symptoms (Maeng et al., 2012). Hakim et al found that HPIV was the third most common viral infections in patients with ALL. Unlike RSV, patients with HPIV in this cohort were more likely to present with URTI (Hakim et al., 2016b). Neutropenia, lymphopenia, coinfection, and use of steroids have been described as risks factors for progression (Srinivasan et al., 2011a,b; Ustun et al., 2012; Seo et al., 2014). LRTI was significantly associated with mortality, between 5 to 31 days from initial diagnosis. The overall mortality rate in this cohort was 23% and no differences were observed when comparing HCT recipients with non-HCT recipients (Maeng et al., 2012). However, other studies reported mortality rates up to 46% (Nichols et al., 2001; Shah et al., 2016b).

There is currently no antiviral approved for the treatment of HPIV. Ribavirin has been used anecdotical in several cases and has not impacted clinical outcomes, including progression to LRTI and mortality (Chemaly et al., 2012; Renaud and Englund, 2012). The use of IVIG has not improved survival of HCT recipients with PIVs infections (Knight et al., 1991; Shah et al., 2012; Chemaly et al., 2014; Waghmare et al., 2016; Shahani et al., 2017).

PIV infection has been linked to bronchiolitis obliterans syndrome (BOS) (Vilchez et al., 2001, 2003; Erard et al., 2006). Treatment alternatives for BOS are limited, in part due to the lack of understanding of its pathogenesis. Furthermore, BOS limits long term survival in these patients, highlighting the importance of prevention of PIVs infection in these patients (Chaparro et al., 1997; Erard et al., 2006).

Although several hospital outbreaks have been reported, nosocomial transmission has been significantly reduced by implementing isolation, hand hygiene, and disinfection of surfaces. There are currently no licensed vaccines for PIVs. Live attenuated PIV vaccines are under development and preliminary results from phase 1 trials suggest that they are safe and immunogenic (Karron et al., 1995a,b; Adderson et al., 2015).

## HMPV

Human metapneumovirus (HMPV) is a single stranded, negative sense RNA enveloped virus from the family *Paramyxoviridae*. The genomic organization is similar to that of RSV with the exception that there is no NS-1 or NS-2. The virus attaches to and enters the host cell through the G and F protein. There are two major subtypes (A and B) composed of subgroups A1/A2 and B1/B2 (Papenburg and Boivin, 2010; Schildgen et al., 2011).

MPV incidence in immunocompromised hosts has been reported to be as high as 40%. The incidence in those with hematological malignancies did not differ compared to HCT recipients. Although no specific risk factor was described in the included studies, incidence in adult patients was slightly higher than in children (Shah et al., 2016c). Hakim et al. found that 10% of all acute respiratory infections with an identified virus in children with ALL was due to HMPV. Of these, only one patient developed LRTI (Hakim et al., 2016b).

HMPV may cause URTI or LRTI in immunocompromised patients. Asymptomatic viral shedding has been described. Patients usually present with nasal congestion, cough, sore throat, or fever. However, once LRTI develops, patients can present with progressive pneumonia, hypotension, and shock (Kamboj et al., 2008; Hoppe et al., 2016; Samuel et al., 2016; Seo et al., 2016; El Chaer et al., 2017).

A recent article of 181 HMPV infections reported that the vast majority of infections are in patients with hematological malignancies or recipients of HCT. Most of the patients had a community acquired infection and 61% presented with URTI. Of those who presented with LRTI, 25% had progression from URTI. The incidence of LRTI in this review was 34% (El Chaer et al., 2017). Risk factors associated with LRTI were use of steroids, low lymphocyte count and, early onset of infection after HCT. LRTI was significantly associated with mortality, which ranged from 0 to 17% in most studies, but can be as high as 43% (Renaud et al., 2013; Shah et al., 2016c; Pasikhova et al., 2018).

There is currently no approved antiviral therapy for HMPV. Several attempts have been made to evaluate the role of antiviral therapy with ribavirin for HMPV infections. Considering the limitations on study design given the low number of patients, most of these studies showed no benefits from antiviral therapy with similar mortality rates when compared patients who did not get antiviral treatment (Hoppe et al., 2016; Shah et al., 2016c; Pasikhova et al., 2018). Fusion inhibitors and monoclonal antibodies are under development but have not yet reached clinical trials (Table 4) (Ulbrandt et al., 2006; Deffrasnes et al., 2008).

## Rhinovirus

The RhVs belong to the genus *Enterovirus* within the family *Picornaviridae*. They include three genetically distinct groups: A, B, and C. They are single-stranded, positive sense, RNA viruses. The genome encodes one polypeptide that is cleaved in four structural proteins (VP1-4) and 7 non-structural proteins. RhVs are antigenically diverse with over 100 serotypes identified to date (Greenberg, 2011; Jacobs et al., 2013).

Rhinovirus has been described as the most common respiratory pathogen isolated in immunocompromised children undergoing HCT (Loria et al., 2015; Fisher et al., 2017), with an incidence as high as 22.3% by day 100 (Milano et al., 2010).

The median time for a first positive tests has been reported at 44 days following HCT but can be as early as 2 days and as late as 93 days. There is no apparent seasonality for RhV detection. Prolonged viral shedding is common with a median of 3 weeks (Milano et al., 2010). Higher viral load at initial presentation has been significantly associated with prolonged viral shedding (Ogimi et al., 2018b). In the former study, thirteen percent of patients with RhV infection had no respiratory symptoms at the time of diagnosis. The remaining patients had URT symptoms. Four patients underwent a BAL for concerns of progression to LRTI, of these two died of respiratory failure (Milano et al., 2010). These results agreed with a study from Seo et al looking at 697 transplant recipients with rhinovirus infection. In this cohort, eighty one percent of patients presented with URTI. Patients with low monocyte count, oxygen use and steroid dose > 1 mg/kg/day had significantly increased risk of LRTI. Mortality was higher in those with LRTI (41 vs. 6%) (Seo et al., 2017). A multicenter study of respiratory viral infections in pediatric HCT recipient reported an all cause fatality rate of 10% in those patients with RhV infection (Fisher et al., 2017).

Pre-transplant rhinovirus detection has been significantly associated with increased mortality at day 100 (Campbell et al., 2015). However, Abandhe et al. did not find any differences in ICU admission, length of hospitalization or mortality (Abandeh et al., 2013). Currently, treatment of RhV infection consists of supportive care. Antiviral medications for RhV are under investigation (Table 4).

## Coronavirus

CoVs are enveloped, positive sense, single stranded RNA viruses belonging to the family *Coronaviridae*. There are six coronaviruses divided in two genera that can infect humans. CoV 229E and NL63 belong to the genus alphacoronavirus and OC43, HKU1, severe acute respiratory syndrome (SARS)-CoV, and Middle East respiratory syndrome (MERS)-CoV are members of the genus betacoronavirus. The genome codes for two non-structural proteins and four structural proteins (van der Hoek, 2007; Greenberg, 2011).

While CoVs are an important cause of the common cold in the general population, there is limited information on the clinical manifestations in immunocompromised hosts. Milano et al. described the epidemiology and risk factors of CoVs infection among HCT recipients. The cumulative incidence estimated at day 100 was 11.1%. The median time of first detection was 53 days (range, 2–93 days). Seasonal outbreaks were common in the winter with 13 of 22 cases first detected in December through March. The median time of shedding was over 3 weeks (Milano et al., 2010).

Ogimi et al. described several risks factors related to prolonged viral shedding, such as higher viral load, use of steroids, and myeloablative conditioning (Ogimi et al., 2017a). Univariate analysis of potential risk factors showed no significant association of acquisition with patient age, gender, underlying disease risk, stem cell source, CMV serostatus, donor type, acute GVHD, conditioning regimen, or engraftment status. Infection with coronaviruses was not associated with mortality in this cohort (Milano et al., 2010). However, another group reported similar mortality rates in HCT recipients to those observed with other viruses such as RSV, influenza virus, and PIV (Ogimi et al., 2017b).

A recent article from Seattle Children's Hospital looked at 85 immunocompromised and 1152 non-immunocompromised children with HCoV infection. Other than median age which was significantly higher for immunocompromised patients, demographic characteristics were similar. Viral co-infection was most commonly seen in non-immunocompromised patients, mostly due to detection of RSV. CoVs strains did not differed between the two groups. The most common clinical presentation was URTI. Younger age, male sex, presence of an underlying pulmonary disorder, and detection of a respiratory co-pathogen, particularly RSV, were associated with an increased likelihood of LRTD or severe LRTD. However, lymphopenia was not associated with more severe disease in this cohort (Ogimi et al., 2018a).

There is currently no treatment available for coronaviruses. However, current *in vitro* evaluation of HCoV therapy includes investigation of antiviral, as well as human monoclonal antibodies (Pyrc et al., 2007; Adedeji et al., 2013) (Table 4).

## New Developments in Therapeutics

### RSV

Nucleoside analogs contain a base, or a base analog linked to a ribose-like moiety that can be incorporated by viral polymerases stopping polymerization and inhibiting replication (Jorquera and Tripp, 2017). ALS-8176 (a.k.a lumicitabine; Alios BioPharma/Janssen) is a nucleoside analog that inhibits the polymerization function of the L protein by causing immediate chain termination of RNA synthesis (Jorquera and Tripp, 2017). In RSV experimentally infected healthy adults, ALS-8176 was significantly associated with more rapid viral clearance, greater reduction of viral load and improvement of clinical symptoms (DeVincenzo et al., 2015). Several fusion inhibitors under development are directed at glycoprotein F with the goal of blocking viral entry into cells lining the respiratory tract (Battles et al., 2016; Jorquera and Tripp, 2017). GS-5806 (a.k.a. Presatovir; Gilead Sciences) is an oral RSV entry inhibitor and functions as an allosteric blocker of the F protein preventing RSV entry by blocking the virus–cell fusion process. Experiments with healthy volunteers with RSV infection showed that treatment with GS-5806 resulted in a reduction of mucus weight, symptom score and viral load (DeVincenzo et al., 2014). Similar results were observed with JNJ-53718678 (Johnson & Johnson) (Stevens et al., 2018). Finally, targeting the nucleoprotein has gained interest given that is highly conserved and essential for virus assembly (Jorquera and Tripp, 2017). RNA interference is a process in which small interfering RNAs (siRNAs) decrease protein production by degrading specific mRNA. Healthy adult volunteers experimentally infected with RSV who received intranasal ALN-RSV01 (Alnylam Pharmaceuticals) showed significant reduction of RSV replication when compared to placebo (DeVincenzo et al., 2010).

Monoclonal (mAbs) have been used for prophylaxis of RSV-related hospital admissions in pediatric populations. There are several mAbs targeting different epitopes of F protein under development (Rossey et al., 2018). REGN2222 (Suptavumab; Regeneron Pharmaceuticals) is a fully human mAb IgG1 that has proven safety and tolerability in healthy adults. In a recent phase III clinical trial REGN222 failed to prevent RSV infections in infants and the company has discontinued further development. MEDI8897 (MedImmune) is a recombinant human IgG1κ mAb with a modified Fc region that extends more than 3-fold its half-life to 85–117 days after one single dose (Zhu Q. et al., 2017). Finally, ALX-0171 (Ablynx) is an inhaled trimeric nanobody that binds the antigenic site II of the F protein and neutralizes RSV A and B. It has shown significant reduction of viral load in infants hospitalized with RSV, when compared to placebo (Ablynx, 2016).

### Influenza

Fludase (DAS181, Ansun BioPharma), a novel sialidase fusion protein, has shown *in vitro* activity toward all influenza strains isolated to date, such as H5N1, H1N1, H7N9 including those that have shown oseltamivir resistance (Triana-Baltzer et al., 2009a,b; Moss et al., 2010). A phase II clinical trial on healthy volunteers showed safety, tolerability, and effectivity in reducing viral load in the groups receiving DAS181 compared to placebo (Moss et al., 2012).

Laninamivir (CS-8958; Biota Pharmaceuticals, Alpharetta, GA, USA) is a long-acting NAI administered via a dry-powder inhaler for the treatment of influenza (Shahani et al., 2017). A double-blind, randomized controlled trial in healthy adults demonstrated that a single inhalation of lanimavir octonate is effective for the treatment of influenza. Importantly, lanimavir remains active against oseltamivir-resistant virus (Watanabe et al., 2010).

Favipiravir (T705; Toyama Chemical, Tokyo, Japan) is an investigational nucleotide analog that inhibits the viral RNA polymerase of most influenza strains (Kiso et al., 2010; Sleeman et al., 2010). Two phase 3 studies on adults with uncomplicated influenza have been completed and results are pending (NCT02026349 and NCT02008344).

JNJ-63623872 (VX-787; Janssen Pharma, Titusville, USA) inhibits influenza A replication by a novel mechanism that involves blocking the activity of the polymerase complex.

Nitazoxanide (NT-300; Romark Laboratories, Florida, USA), an antiparasitic agent, has shown broad activity toward influenza A including A(pH1N1) and H7N9, as well as influenza B and strains resistant to oseltamivir (Rossignol, 2014; Tilmanis et al., 2017). A Phase 2b/3 clinical trial comparing nitazoxanide vs. placebo, showed reduction of clinical symptoms and viral shedding in patients with uncomplicated influenza infection (Haffizulla et al., 2014). There are several ongoing trials including a phase 3 to evaluate the role of nitazoxanide for children 12 year of age or older and adults with uncomplicated influenza infection (NCT03336619). mAbs directed toward the highly conserved HA stalk of influenza virus are being developed for both prophylaxis and treatment (Shahani et al., 2017).

### Parainfluenza

Paradase (DAS181, Ansun BioPharma) is a novel sialidase fusion protein that prevents viral propagation by cleaving sialic acid from respiratory epithelial cells leading to impair entry of the virus to the cells (Guzmán-Suarez et al., 2012; Chalkias et al., 2014; Chemaly et al., 2014). DAS181 has been given for compassionate use in immunocompromised patients, and has been associated with benefits (Chen et al., 2011; Drozd et al., 2013; Waghmare et al., 2015). Ansun Biopharma has completed a multi-site phase II study comparing DAS181 with placebo in immunocompromised patients with Parainfluenza LRTI requiring oxygen (NCT01644877). In 2017 the U.S. Food and Drug Administration (FDA) has granted Breakthrough Therapy Designation to DAS181 for the treatment of lower respiratory tract PIV infection in immunocompromised patients (Biopharma, 2017). Phase 3 trials are currently being planned.

### Metapneumovirus

Fusion inhibitors targeting the F protein have demonstrated viral inhibition *in vitro* and in animal models (Deffrasnes et al., 2008). A phase 2b study to evaluate the safety, tolerability and pharmacokintetics of ALS-8176 (a.k.a lumicitabine; Alios BioPharma/Janssen) in hospitalized adults with metapneumovirus infection is being planned (NCT03502694). Two mAbs, MAb 338 (Medimmune, Gaithersburg, MD, USA) and human Fab DS7, have prophylactic and therapeutic potential (Ulbrandt et al., 2006). However, clinical studies have not yet been performed.

### Rhinovirus

Vapendavir (Aviragen Therapeutics, Alpharetta, GA, USA) is an oral antiviral that attaches to the viral capsid preventing release of RNA into the cells. It has activity against RhV type A and B (Shahani et al., 2017). Vapendavir has reduced clinical symptoms in asthmatic adults. It was well-tolerated and there were no serious adverse events reported (Matz, 2013).

### Coronavirus

Treatment of coronavirus will require broadly active compounds designed to quickly respond to new zoonotic epidemics of pathogenic coronaviruses. There are several steps in coronavirus life cycle where antiviral agents could be implemented (Pyrc et al., 2007). Adedeji et al. reported different strategies under development to block viral entry of SARS-CoV (Adedeji et al., 2013).

### Adoptive T Cell Therapy

Adoptive transfer of specific T cells is a promising approach as technologies for isolation and expansion become widely available (Englund et al., 2011; Gerdemann et al., 2012; Heslop and Leen, 2013; Leen et al., 2014; Baugh et al., 2018). There are two main limitations to immunotherapy strategies for respiratory viruses. One is that, with the exception of influenza, knowledge of viral immunology is less complete than for CMV and EBV. A second limitation is logistic in that these viruses cause the greatest morbidity in the early post transplantation period, and it may not be feasible to generate CTLs in time to cover this period of high risk. However, Aguayo-Hiraldo et al. successfully expanded and use PIV specific T cells (Aguayo-Hiraldo et al., 2017). The same group generated HMPV specific T cells for adoptive transfer in patients after allogeneic HCT (Tzannou et al., 2017). Finally, multivirus-specific lymphocytes for the RSV and Influenza have shown promising results (Gerdemann et al., 2012).

## Conclusion

Immunocompromised patients remain vulnerable to respiratory viral infections. Risk of severe LRTI and increased mortality are a common characteristic. Hematological malignancies and HCT recipients bear most of the burden of these viruses. Age, baseline diagnosis, use of steroids, lymphopenia, and early onset after transplant are shared risk factors for LRTI and mortality. Higher viral load has been associated with risk of LRTI in some studies. Early diagnosis can help prevent further spread of these viruses and prompt supportive measures and antivirals when indicated. However, management of these infections remains challenging. Clinical symptoms are not always reliable and progression to LRTI can happen within days of onset of symptoms. In addition, most patients have prolonged viral shedding, making both clinical evolution and molecular testing insufficient to guide length of therapy. Persistently positive tests often lead to concerns of resistant viral strains given the high within host viral diversification that occurs in these patients. There are limited antiviral options available mostly for influenza and to some extent RSV. These problems highlight the need for objective biomarkers that could help guide therapy. Standardization of valid assays to assess viral load will provide unbiased and reproducible measures that could be universally applied to the management of respiratory viral infections in immunocompromised patients. Immune monitoring assays assessing IFN-**γ** producing T cells against specific antigens are gaining interest in the field of systemic viral infection. A set of objective and validated tools related to the host-viral interaction (such as viral load and specific immune response) could lead to parameters that will significantly impact decision making on how to approach and monitor therapeutic options in immunocompromised patients with respiratory viral infections. Having early objective predictors of adverse outcomes could identify children at highest risk for LRTI and death, directing treatment more specifically and in a timelier manner than is currently possible with potential benefits of increased pediatric survival.

## Author Contributions

DH performed the search and literature review. DH, GM, and RH wrote the manuscript. All authors approved the final version of the manuscript.

### Conflict of Interest Statement

RH has served on advisory boards for Roche Molecular, Abbott molecular, and Quidel. The remaining authors declare that the research was conducted in the absence of any commercial or financial relationships that could be construed as a potential conflict of interest.

## References

[B1] AAP (2014a). Updated guidance for palivizumab prophylaxis among infants and young children at increased risk of hospitalization for respiratory syncytial virus infection. Pediatrics 134, e620–e638. 10.1542/peds.2014-1666 25070304

[B2] AAP (2014b). Updated guidance for palivizumab prophylaxis among infants and young children at increased risk of hospitalization for respiratory syncytial virus infection. Pediatrics 134, 415–420. 10.1542/peds.2014-1665 25070315

[B3] AAP (2018). Recommendations for prevention and control of influenza in children, 2018–2019. Pediatrics 140:e20172550 10.1542/peds.2017-2550

[B4] Abandeh F. I. Lustberg M. Devine S. Elder P. Andritsos L. Martin S. I. (2013). Outcomes of hematopoietic stem cell transplant recipients with rhinovirus infection: a matched, case-control study. Bone Marrow Transplant. 48, 1554–1557. 10.1038/bmt.2013.100 23872740 PMC4606879

[B5] Ablynx (2016). Inhaled ALX-0171: Phase I/IIa Study in 53 hospitalised RSV-Infected Children [Online]. Available online at: http://www.ablynx.com/uploads/data/files/ablynx_alx-0171_first-in-infant%20study%20results_webcast%20presentation.pdf [Accessed].

[B6] Adderson E. Branum K. Sealy R. E. Jones B. G. Surman S. L. Penkert R.. (2015). Safety and immunogenicity of an intranasal Sendai virus-based human parainfluenza virus type 1 vaccine in 3- to 6-year-old children. Clin. Vaccine Immunol. 22, 298–303. 10.1128/cvi.00618-14 25552633 PMC4340902

[B7] Adedeji A. O. Severson W. Jonsson C. Singh K. Weiss S. R. Sarafianos S. G. (2013). Novel inhibitors of severe acute respiratory syndrome coronavirus entry that act by three distinct mechanisms. J. Virol. 87, 8017–8028. 10.1128/jvi.00998-13 23678171 PMC3700180

[B8] Aguayo-Hiraldo P. I. Arasaratnam R. J. Tzannou I. Kuvalekar M. Lulla P. Naik S.. (2017). Characterizing the cellular immune response to parainfluenza virus 3. J. Infect. Dis. 216, 153–161. 10.1093/infdis/jix203 28472480 PMC5853958

[B9] Ambrose C. S. McLaurin K. K. (2015). The medicaid cost of palivizumab. J. Pediatr. Infect. Dis. Soc. 4, 83–84. 10.1093/jpids/piu025 25844167 PMC4381742

[B10] Appiah G. D. Blanton L. D'Mello T. Kniss K. Smith S. Mustaquim D.. (2015). Influenza activity - United States, 2014-15 season and composition of the 2015-16 influenza vaccine. MMWR Morb. Mortal. Wkly. Rep. 64, 583–590. 26042650 PMC4584770

[B11] Azadeh N. Sakata K. K. Brighton A. M. Vikram H. R. Grys T. E. (2015). FilmArray respiratory panel assay: comparison of nasopharyngeal swabs and bronchoalveolar lavage samples. J. Clin. Microbiol. 53, 3784–3787. 10.1128/jcm.01516-15 26378282 PMC4652125

[B12] Battles M. B. Langedijk J. P. Furmanova-Hollenstein P. Chaiwatpongsakorn S. Costello H. M. Kwanten L.. (2016). Molecular mechanism of respiratory syncytial virus fusion inhibitors. Nat. Chem. Biol. 12, 87–93. 10.1038/nchembio.1982 26641933 PMC4731865

[B13] Baugh K. A. Tzannou I. Leen A. M. (2018). Infusion of cytotoxic T lymphocytes for the treatment of viral infections in hematopoetic stem cell transplant patients. Curr. Opin. Infect. Dis. 31, 292–300. 10.1097/qco.0000000000000456 29750672 PMC7896200

[B14] Beck C. R. McKenzie B. C. Hashim A. B. Harris R. C. Nguyen-Van-Tam J. S. (2012). Influenza vaccination for immunocompromised patients: systematic review and meta-analysis by etiology. J. Infect. Dis. 206, 1250–1259. 10.1093/infdis/jis487 22904335

[B15] Bednarska K. Hallmann-Szelinska E. Kondratiuk K. Brydak L. B. (2015). Evaluation of the activity of influenza and influenza-like viruses in the epidemic season 2013/2014. Adv. Exp. Med. Biol. 857, 1–7. 10.1007/5584_2015_116 25786402 PMC7121991

[B16] Biopharma A. (2017). Ansun BioPharma Announces Breakthrough Designation for Its Experimental Drug DAS181 [Online]. Available online at: http://www.ansunbiopharma.com/news/ (Accessed September 3, 2018).

[B17] Boeckh M. Englund J. Li Y. Miller C. Cross A. Fernandez H.. (2007). Randomized controlled multicenter trial of aerosolized ribavirin for respiratory syncytial virus upper respiratory tract infection in hematopoietic cell transplant recipients. Clin. Infect. Dis. 44, 245–249. 10.1086/509930 17173225

[B18] Boeckh M. Englund J. A. (2010). Evaluation of therapeutics for RSV: an important step forward. Am. J. Respir. Crit. Care Med. 182, 1217–1219. 10.1164/rccm.201008-1230ED 21079265

[B19] Buckingham S. C. Bush A. J. Devincenzo J. P. (2000). Nasal quantity of respiratory syncytical virus correlates with disease severity in hospitalized infants. Pediatr. Infect. Dis. J. 19, 113–117. 10.1097/00006454-200002000-00006 10693996

[B20] Byrn R. A. Jones S. M. Bennett H. B. Bral C. Clark M. P. Jacobs M. D.. (2015). Preclinical activity of VX-787, a first-in-class, orally bioavailable inhibitor of the influenza virus polymerase PB2 subunit. Antimicrob. Agents Chemother. 59, 1569–1582. 10.1128/aac.04623-14 25547360 PMC4325764

[B21] Caliendo A. M. (2011). Multiplex PCR and emerging technologies for the detection of respiratory pathogens. Clin. Infect. Dis. 52(Suppl. 4), S326–S330. 10.1093/cid/cir047 21460291 PMC7107927

[B22] Campbell A. P. Guthrie K. A. Englund J. A. Farney R. M. Minerich E. L. Kuypers J.. (2015). Clinical outcomes associated with respiratory virus detection before allogeneic hematopoietic stem cell transplant. Clin. Infect. Dis. 61, 192–202. 10.1093/cid/civ272 25847977 PMC4565994

[B23] Capella C. Chaiwatpongsakorn S. Gorrell E. Risch Z. A. Ye F. Mertz S. E.. (2017). Prefusion F, postfusion F, G antibodies, and disease severity in infants and young children with acute respiratory syncytial virus infection. J. Infect. Dis. 216, 1398–1406. 10.1093/infdis/jix489 29029312 PMC5853469

[B24] Carr S. Allison K. J. Van De Velde L. A. Zhang K. English E. Y. Iverson A.. (2011a). Safety and immunogenicity of live attenuated and inactivated influenza vaccines in children with cancer. J. Infect. Dis. 204, 1475–1482. 10.1093/infdis/jir561 21949042

[B25] Carr S. Ilyushina N. A. Franks J. Adderson E. E. Caniza M. Govorkova E. A.. (2011b). Oseltamivir-resistant influenza A and B viruses pre- and postantiviral therapy in children and young adults with cancer. Pediatr. Infect. Dis. J. 30, 284–288. 10.1097/INF.0b013e3181ff863b 21048522 PMC3070406

[B26] Caserta M. T. Qiu X. Tesini B. Wang L. Murphy A. Corbett A.. (2017). Development of a global respiratory severity score for respiratory syncytial virus infection in infants. J. Infect. Dis. 215, 750–756. 10.1093/infdis/jiw624 28011907 PMC5388274

[B27] Casper C. Englund J. Boeckh M. (2010). How I treat influenza in patients with hematologic malignancies. Blood 115, 1331–1342. 10.1182/blood-2009-11-255455 20009037 PMC2826758

[B28] Chalkias S. Mackenzie M. R. Gay C. Dooley C. Marty F. M. Moss R. B.. (2014). DAS181 treatment of hematopoietic stem cell transplant patients with parainfluenza virus lung disease requiring mechanical ventilation. Transpl. Infect. Dis. 16, 141–144. 10.1111/tid.12177 24383501 PMC4469988

[B29] Chaparro C. Scavuzzo M. Winton T. Keshavjee S. Kesten S. (1997). Status of lung transplant recipients surviving beyond five years. J. Heart Lung Transplant. 16, 511–516. 9171269

[B30] Chemaly R. F. Aitken S. L. Wolfe C. R. Jain R. Boeckh M. J. (2016). Aerosolized ribavirin: the most expensive drug for pneumonia. Transpl. Infect. Dis. 18, 634–636. 10.1111/tid.12551 27214684

[B31] Chemaly R. F. Ghosh S. Bodey G. P. Rohatgi N. Safdar A. Keating M. J.. (2006). Respiratory viral infections in adults with hematologic malignancies and human stem cell transplantation recipients: a retrospective study at a major cancer center. Medicine 85, 278–287. 10.1097/01.md.0000232560.22098.4e 16974212

[B32] Chemaly R. F. Hanmod S. S. Rathod D. B. Ghantoji S. S. Jiang Y. Doshi A. (2012). The characteristics and outcomes of parainfluenza virus infections in 200 patients with leukemia or recipients of hematopoietic stem cell transplantation. Blood 119, 2738–2745; quiz 2969. 10.1182/blood-2011-08-371112 22246027

[B33] Chemaly R. F. Shah D. P. Boeckh M. J. (2014). Management of respiratory viral infections in hematopoietic cell transplant recipients and patients with hematologic malignancies. Clin. Infect. Dis. 59(Suppl. 5), S344–S351. 10.1093/cid/ciu623 25352629 PMC4303052

[B34] Chen Y. B. Driscoll J. P. McAfee S. L. Spitzer T. R. Rosenberg E. S. Sanders R.. (2011). Treatment of parainfluenza 3 infection with DAS181 in a patient after allogeneic stem cell transplantation. Clin. Infect. Dis. 53, e77–e80. 10.1093/cid/cir501 21880586

[B35] Choi S. M. Boudreault A. A. Xie H. Englund J. A. Corey L. Boeckh M. (2011). Differences in clinical outcomes after 2009 influenza A/H1N1 and seasonal influenza among hematopoietic cell transplant recipients. Blood 117, 5050–5056. 10.1182/blood-2010-11-319186 21372154 PMC3109531

[B36] Chu H. Y. Chin J. Pollard J. Zerr D. M. Englund J. A. (2016). Clinical outcomes in outpatient respiratory syncytial virus infection in immunocompromised children. Influenza Other Respir. Viruses 10, 205–210. 10.1111/irv.12375 26859306 PMC4814860

[B37] Clark M. P. Ledeboer M. W. Davies I. Byrn R. A. Jones S. M. Perola E.. (2014). Discovery of a novel, first-in-class, orally bioavailable azaindole inhibitor (VX-787) of influenza PB2. J. Med. Chem. 57, 6668–6678. 10.1021/jm5007275 25019388

[B38] Coates M. Brookes D. Kim Y. I. Allen H. Fordyce E. A. F. Meals E. A.. (2017). Preclinical characterization of PC786, an inhaled small-molecule respiratory syncytial virus L protein polymerase inhibitor. Antimicrob. Agents Chemother. 61:e00737–17. 10.1128/aac.00737-17 28652242 PMC5571287

[B39] Couch R. B. Englund J. A. Whimbey E. (1997). Respiratory viral infections in immunocompetent and immunocompromised persons. Am. J. Med. 102, 2–9; discussion 25–26. 10868136 10.1016/S0002-9343(97)00003-X PMC7124320

[B40] Couturier B. A. Bender J. M. Schwarz M. A. Pavia A. T. Hanson K. E. She R. C. (2010). Oseltamivir-resistant influenza A 2009 H1N1 virus in immunocompromised patients. Influenza Other Respir. Viruses 4, 199–204. 10.1111/j.1750-2659.2010.00144.x 20836794 PMC5964545

[B41] Damlaj M. Bartoo G. Gijima D. Hashmi S. Litzow M. R. Hogan W. (2015). Oral ribavirin for the treatment of respiratory syncytial virus infection in allogeneic stem cell transplant recipients-utility of the immunodeficiency scoring index for risk stratification. Biol. Blood Marrow Transplant. 21, S173–S174. 10.1016/j.bbmt.2014.11.255

[B42] de Fontbrune F. S. Robin M. Porcher R. Scieux C. de Latour R. P. Ferry C.. (2007). Palivizumab treatment of respiratory syncytial virus infection after allogeneic hematopoietic stem cell transplantation. Clin. Infect. Dis. 45, 1019–1024. 10.1086/521912 17879919

[B43] de Jong M. D. Ison M. G. Monto A. S. Metev H. Clark C. O'Neil B.. (2014). Evaluation of intravenous peramivir for treatment of influenza in hospitalized patients. Clin. Infect. Dis. 59, e172–e185. 10.1093/cid/ciu632 25115871

[B44] Deffrasnes C. Hamelin M. E. Prince G. A. Boivin G. (2008). Identification and evaluation of a highly effective fusion inhibitor for human metapneumovirus. Antimicrob. Agents Chemother. 52, 279–287. 10.1128/aac.00793-07 17967906 PMC2223880

[B45] DeVincenzo J. Lambkin-Williams R. Wilkinson T. Cehelsky J. Nochur S. Walsh E.. (2010). A randomized, double-blind, placebo-controlled study of an RNAi-based therapy directed against respiratory syncytial virus. Proc. Natl. Acad. Sci. U.S.A. 107, 8800–8805. 10.1073/pnas.0912186107 20421463 PMC2889365

[B46] Devincenzo J. P. (2004). Natural infection of infants with respiratory syncytial virus subgroups A and B: a study of frequency, disease severity, and viral load. Pediatr. Res. 56, 914–917. 10.1203/01.Pdr.0000145255.86117.6a 15470202

[B47] DeVincenzo J. P. Aitken J. Harrison L. (2003). Respiratory syncytial virus (RSV) loads in premature infants with and without prophylactic RSV fusion protein monoclonal antibody. J. Pediatr. 143, 123–126. 10.1016/s0022-3476(03)00213-0 12915838

[B48] DeVincenzo J. P. Buckingham S. C. (2002). Relationship between respiratory syncytial virus load and illness severity in children. J. Infect. Dis. 186, 1376–1377; author reply 1377. 10.1086/344331 12402213

[B49] DeVincenzo J. P. El Saleeby C. M. Bush A. J. (2005). Respiratory syncytial virus load predicts disease severity in previously healthy infants. J. Infect. Dis. 191, 1861–1868. 10.1086/430008 15871119

[B50] DeVincenzo J. P. McClure M. W. Symons J. A. Fathi H. Westland C. Chanda S.. (2015). Activity of oral ALS-008176 in a respiratory syncytial virus challenge study. N. Engl. J. Med. 373, 2048–2058. 10.1056/NEJMoa1413275 26580997

[B51] DeVincenzo J. P. Whitley R. J. Mackman R. L. Scaglioni-Weinlich C. Harrison L. Farrell E.. (2014). Oral GS-5806 activity in a respiratory syncytial virus challenge study. N. Engl. J. Med. 371, 711–722. 10.1056/NEJMoa1401184 25140957

[B52] Domachowske J. B. Rosenberg H. F. (1999). Respiratory syncytial virus infection: immune response, immunopathogenesis, and treatment. Clin. Microbiol. Rev. 12, 298–309. 10194461 10.1128/cmr.12.2.298 PMC88919

[B53] Douglas J. L. Panis M. L. Ho E. Lin K. Y. Krawczyk S. H. Grant D. M.. (2005). Small molecules VP-14637 and JNJ-2408068 inhibit respiratory syncytial virus fusion by similar mechanisms. Antimicrob. Agents Chemother. 49, 2460–2466. 10.1128/aac.49.6.2460-2466.2005 15917547 PMC1140497

[B54] Drozd D. R. Limaye A. P. Moss R. B. Sanders R. L. Hansen C. Edelman J. D.. (2013). DAS181 treatment of severe parainfluenza type 3 pneumonia in a lung transplant recipient. Transpl. Infect. Dis. 15, E28–32. 10.1111/tid.12045 23279859 PMC7169673

[B55] Dykewicz C. A. (2001). Guidelines for preventing opportunistic infections among hematopoietic stem cell transplant recipients: focus on community respiratory virus infections. Biol. Blood Marrow Transplant. 7(Suppl.), 19s–22s. 10.1053/bbmt.2001.v7.pm11777100 11777100

[B56] El Chaer F. Shah D. P. Kmeid J. Ariza-Heredia E. J. Hosing C. M. Mulanovich V. E.. (2017). Burden of human metapneumovirus infections in patients with cancer: risk factors and outcomes. Cancer 123, 2329–2337. 10.1002/cncr.30599 28178369 PMC5459658

[B57] El Saleeby C. M. Bush A. J. Harrison L. M. Aitken J. A. Devincenzo J. P. (2011). Respiratory syncytial virus load, viral dynamics, and disease severity in previously healthy naturally infected children. J. Infect. Dis. 204, 996–1002. 10.1093/infdis/jir494 21881113 PMC3203391

[B58] El Saleeby C. M. Somes G. W. DeVincenzo J. P. Gaur A. H. (2008). Risk factors for severe respiratory syncytial virus disease in children with cancer: the importance of lymphopenia and young age. Pediatrics 121, 235–243. 10.1542/peds.2007-1102 18245413

[B59] Engelhard D. Mohty B. de la Camara R. Cordonnier C. Ljungman P. (2013). European guidelines for prevention and management of influenza in hematopoietic stem cell transplantation and leukemia patients: summary of ECIL-4 (2011), on behalf of ECIL, a joint venture of EBMT, EORTC, ICHS, and ELN. Transpl. Infect. Dis. 15, 219–232. 10.1111/tid.12054 23363310

[B60] Engelhard D. Nagler A. Hardan I. Morag A. Aker M. Baciu H.. (1993). Antibody response to a two-dose regimen of influenza vaccine in allogeneic T cell-depleted and autologous BMT recipients. Bone Marrow Transplant. 11, 1–5. 8431706

[B61] Englund J. Feuchtinger T. Ljungman P. (2011). Viral infections in immunocompromised patients. Biol. Blood Marrow Transplant. 17, S2–S5. 10.1016/j.bbmt.2010.11.008 21195305 PMC3030455

[B62] Englund J. A. (2001). Diagnosis and epidemiology of community-acquired respiratory virus infections in the immunocompromised host. Biol. Blood Marrow Transplant. 7(Suppl.), 2s–4s. 10.1053/bbmt.2001.v7.pm11777101 11777101

[B63] Englund J. A. Piedra P. A. Ahn Y. M. Gilbert B. E. Hiatt P. (1994). High-dose, short-duration ribavirin aerosol therapy compared with standard ribavirin therapy in children with suspected respiratory syncytial virus infection. J. Pediatr. 125, 635–641. 7931890 10.1016/s0022-3476(94)70026-5

[B64] Englund J. A. Piedra P. A. Jefferson L. S. Wilson S. Z. Taber L. H. Gilbert B. E. (1990). High-dose, short-duration ribavirin aerosol therapy in children with suspected respiratory syncytial virus infection. J. Pediatr. 117(2 Pt 1), 313–320. 2380833 10.1016/s0022-3476(05)80554-2

[B65] Englund J. A. Piedra P. A. Jewell A. Patel K. Baxter B. B. Whimbey E. (1996). Rapid diagnosis of respiratory syncytial virus infections in immunocompromised adults. J. Clin. Microbiol. 34, 1649–1653. 8784563 10.1128/jcm.34.7.1649-1653.1996 PMC229088

[B66] Englund J. A. Whimbey E. Atmar R. L. (1999). Diagnosis of respiratory viruses in cancer and transplant patients. Curr. Clin. Top. Infect. Dis. 19, 30–59. 10472479

[B67] Erard V. Chien J. W. Kim H. W. Nichols W. G. Flowers M. E. Martin P. J.. (2006). Airflow decline after myeloablative allogeneic hematopoietic cell transplantation: the role of community respiratory viruses. J. Infect. Dis. 193, 1619–1625. 10.1086/504268 16703503 PMC7110078

[B68] Espinosa-Aguilar L. Green J. S. Forrest G. N. Ball E. D. Maziarz R. T. Strasfeld L.. (2011). Novel H1N1 influenza in hematopoietic stem cell transplantation recipients: two centers' experiences. Biol. Blood Marrow Transplant. 17, 566–573. 10.1016/j.bbmt.2010.07.018 20708084

[B69] Falsey A. R. Koval C. DeVincenzo J. P. Walsh E. E. (2017). Compassionate use experience with high-titer respiratory syncytical virus (RSV) immunoglobulin in RSV-infected immunocompromised persons. Transpl. Infect. Dis. 19:e12657. 10.1111/tid.12657 28054734

[B70] FDA (2018). FDA Approves New Drug to Treat Influenza [Online]. U.S. Food and Drug Administration. Available online at: https://www.fda.gov/NewsEvents/Newsroom/PressAnnouncements/ucm624226.htm (Accessed October 25, 2018).

[B71] Fiore A. E. Fry A. Shay D. Gubareva L. Bresee J. S. Uyeki T. M. (2011). Antiviral agents for the treatment and chemoprophylaxis of influenza — recommendations of the Advisory Committee on Immunization Practices (ACIP). MMWR Recomm. Rep. 60, 1–24. 21248682

[B72] Fisher B. T. Danziger-Isakov L. Sweet L. R. Munoz F. M. Maron G. Tuomanen E. (2017). A multicenter consortium to define the epidemiology and outcomes of inpatient respiratory viral infections in pediatric hematopoietic stem cell transplant recipients. J. Pediatric Infect. Dis. Soc. 7, 275–282. 10.1093/jpids/pix051 PMC7107490 29106589

[B73] Foolad F. Aitken S. L. Shigle T. L. Prayag A. Ghantoji S. S. Ariza-Heredia E. J. (2018). Impact of ribavirin formulation and host risk factors on 90-day mortality in Hematopoietic Cell Transplant (HCT) patients with RSV infection. Biol. Blood Marrow Transplant. 24, S369–S370. 10.1016/j.bbmt.2017.12.451

[B74] Foolad F. Prayag A. Ghantoji S. S. Ariza-Heredia E. J. Chemaly R. F. (2017). Use of oral ribavirin for the treatment of RSV infections in Hematopoietic Cell Transplant (HCT) recipients. Biol. Blood Marrow Transplant. 23:S189 10.1016/j.bbmt.2016.12.367

[B75] Fraaij P. L. Schutten M. Javouhey E. Burleigh L. Outlaw R. Kumar D.. (2015). Viral shedding and susceptibility to oseltamivir in hospitalized immunocompromised patients with influenza in the Influenza Resistance Information Study (IRIS). Antivir. Ther. 20, 633–642. 10.3851/imp2957 25849228

[B76] Franz A. Adams O. Willems R. Bonzel L. Neuhausen N. Schweizer-Krantz S.. (2010). Correlation of viral load of respiratory pathogens and co-infections with disease severity in children hospitalized for lower respiratory tract infection. J. Clin. Virol. 48, 239–245. 10.1016/j.jcv.2010.05.007 20646956 PMC7185496

[B77] Fuller J. A. Njenga M. K. Bigogo G. Aura B. Ope M. O. Nderitu L.. (2013). Association of the CT values of real-time PCR of viral upper respiratory tract infection with clinical severity, Kenya. J. Med. Virol. 85, 924–932. 10.1002/jmv.23455 23508918

[B78] Gerdemann U. Keirnan J. M. Katari U. L. Yanagisawa R. Christin A. S. Huye L. E.. (2012). Rapidly generated multivirus-specific cytotoxic T lymphocytes for the prophylaxis and treatment of viral infections. Mol. Ther. 20, 1622–1632. 10.1038/mt.2012.130 22801446 PMC3412490

[B79] Gerna G. Campanini G. Rognoni V. Marchi A. Rovida F. Piralla A.. (2008). Correlation of viral load as determined by real-time RT-PCR and clinical characteristics of respiratory syncytial virus lower respiratory tract infections in early infancy. J. Clin. Virol. 41, 45–48. 10.1016/j.jcv.2007.10.018 18082444

[B80] Ghosh S. Champlin R. E. Englund J. Giralt S. A. Rolston K. Raad I.. (2000). Respiratory syncytial virus upper respiratory tract illnesses in adult blood and marrow transplant recipients: combination therapy with aerosolized ribavirin and intravenous immunoglobulin. Bone Marrow Transplant. 25, 751–755. 10.1038/sj.bmt.1702228 10745261

[B81] Gilchrist C. A. Turner S. D. Riley M. F. Petri W. A. Jr. Hewlett E. L. (2015). Whole-genome sequencing in outbreak analysis. Clin. Microbiol. Rev. 28, 541–563. 10.1128/cmr.00075-13 25876885 PMC4399107

[B82] Greenberg S. B. (2011). Update on rhinovirus and coronavirus infections. Semin. Respir. Crit. Care Med. 32, 433–446. 10.1055/s-0031-1283283 21858748

[B83] Greninger A. L. Zerr D. M. Qin X. Adler A. L. Sampoleo R. Kuypers J. M.. (2017). Rapid metagenomic next-generation sequencing during an investigation of hospital-acquired human parainfluenza virus 3 infections. J. Clin. Microbiol. 55, 177–182. 10.1128/jcm.01881-16 27795347 PMC5228228

[B84] Gruber W. C. Wilson S. Z. Throop B. J. Wyde P. R. (1987). Immunoglobulin administration and ribavirin therapy: efficacy in respiratory syncytial virus infection of the cotton rat. Pediatr. Res. 21, 270–274. 10.1203/00006450-198703000-00013 3550674

[B85] Guzmán-Suarez B. B. Buckley M. W. Gilmore E. T. Vocca E. Moss R. Marty F. M.. (2012). Clinical potential of DAS181 for treatment of parainfluenza-3 infections in transplant recipients. Transpl. Infect. Dis. 14, 427–433. 10.1111/j.1399-3062.2012.00718.x 22340538

[B86] Haffizulla J. Hartman A. Hoppers M. Resnick H. Samudrala S. Ginocchio C.. (2014). Effect of nitazoxanide in adults and adolescents with acute uncomplicated influenza: a double-blind, randomised, placebo-controlled, phase 2b/3 trial. Lancet Infect. Dis. 14, 609–618. 10.1016/s1473-3099(14)70717-0 24852376 PMC7164783

[B87] Hakim H. Allison K. J. Van de Velde L. A. Tang L. Sun Y. Flynn P. M. (2016a). Immunogenicity and safety of high-dose trivalent inactivated influenza vaccine compared to standard-dose vaccine in children and young adults with cancer or HIV infection. Vaccine 34, 3141–3148. 10.1016/j.vaccine.2016.04.053 27129426 PMC4899146

[B88] Hakim H. Dallas R. Zhou Y. Pei D. Cheng C. Flynn P. M.. (2016b). Acute respiratory infections in children and adolescents with acute lymphoblastic leukemia. Cancer 122, 798–805. 10.1002/cncr.29833 26700662 PMC4764417

[B89] Hakki M. Strasfeld L. M. Townes J. M. (2014). Predictive value of testing nasopharyngeal samples for respiratory viruses in the setting of lower respiratory tract disease. J. Clin. Microbiol. 52, 4020–4022. 10.1128/jcm.01944-14 25122864 PMC4313242

[B90] Hall C. B. (2001). Respiratory syncytial virus and parainfluenza virus. N. Engl. J. Med. 344, 1917–1928. 10.1056/nejm200106213442507 11419430

[B91] Hall C. B. Simoes E. A. Anderson L. J. (2013). Clinical and epidemiologic features of respiratory syncytial virus. Curr. Top. Microbiol. Immunol. 372, 39–57. 10.1007/978-3-642-38919-1_2 24362683

[B92] Hall C. B. Walsh E. E. Schnabel K. C. Long C. E. McConnochie K. M. Hildreth S. W.. (1990). Occurrence of groups A and B of respiratory syncytial virus over 15 years: associated epidemiologic and clinical characteristics in hospitalized and ambulatory children. J. Infect. Dis. 162, 1283–1290. 2230258 10.1093/infdis/162.6.1283

[B93] Hall C. B. Weinberg G. A. Iwane M. K. Blumkin A. K. Edwards K. M. Staat M. A.. (2009). The burden of respiratory syncytial virus infection in young children. N. Engl. J. Med. 360, 588–598. 10.1056/NEJMoa0804877 19196675 PMC4829966

[B94] Hamada N. Imamura Y. Hara K. Kashiwagi T. Imamura Y. Nakazono Y.. (2012). Intrahost emergent dynamics of oseltamivir-resistant virus of pandemic influenza A (H1N1) 2009 in a fatally immunocompromised patient. J. Infect. Chemother. 18, 865–871. 10.1007/s10156-012-0429-0 22661221 PMC7101931

[B95] Hammond S. P. Gagne L. S. Stock S. R. Marty F. M. Gelman R. S. Marasco W. A.. (2012). Respiratory virus detection in immunocompromised patients with FilmArray respiratory panel compared to conventional methods. J. Clin. Microbiol. 50, 3216–3221. 10.1128/jcm.00538-12 22814461 PMC3457462

[B96] Hayden F. G. Sugaya N. Hirotsu N. Lee N. de Jong M. D. Hurt A. C.. (2018). Baloxavir marboxil for uncomplicated influenza in adults and adolescents. N. Engl. J. Med. 379, 913–923. 10.1056/NEJMoa1716197 30184455

[B97] Hayden R. T. Gu Z. Rodriguez A. Tanioka L. Ying C. Morgenstern M.. (2012). Comparison of two broadly multiplexed PCR systems for viral detection in clinical respiratory tract specimens from immunocompromised children. J. Clin. Virol. 53, 308–313. 10.1016/j.jcv.2011.12.020 22296791 PMC7108354

[B98] Henrickson K. J. (2003). Parainfluenza viruses. Clin. Microbiol. Rev. 16, 242–264. 10.1128/CMR.16.2.242-264.2003 12692097 PMC153148

[B99] Heo Y. A. (2018). Baloxavir: first global approval. Drugs 78, 693–697. 10.1007/s40265-018-0899-1 29623652

[B100] Heslop H. E. Leen A. M. (2013). T-cell therapy for viral infections. Hematol. Am. Soc. Hematol. Educ. Program 2013, 342–347. 10.1182/asheducation-2013.1.342 24319202

[B101] Heylen E. Neyts J. Jochmans D. (2017). Drug candidates and model systems in respiratory syncytial virus antiviral drug discovery. Biochem. Pharmacol. 127, 1–12. 10.1016/j.bcp.2016.09.014 27659812

[B102] Hirsch H. H. Martino R. Ward K. N. Boeckh M. Einsele H. Ljungman P. (2013). Fourth European Conference on Infections in Leukaemia (ECIL-4): guidelines for diagnosis and treatment of human respiratory syncytial virus, parainfluenza virus, metapneumovirus, rhinovirus, and coronavirus. Clin. Infect. Dis. 56, 258–266. 10.1093/cid/cis844 23024295 PMC3526251

[B103] Hodinka R. L. (2016). “Respiratory RNA Viruses,” in Diagnostic Microbiology of the Immunocompromised Host, 2nd Edn, eds Hayden R. T. Wolk D. M. Carroll K. C. Tang Y.-W. (Washington, DC: American Society of Microbiology), 233–271.

[B104] Hoppe B. P. de Jongh E. Griffioen-Keijzer A. Zijlstra-Baalbergen J. M. IJzerman E. P. Baboe F. (2016). Human metapneumovirus in haematopoietic stem cell transplantation recipients: a case series and review of the diagnostic and therapeutic approach. Neth. J. Med. 74, 336–341. 27762221

[B105] Houlihan C. F. Frampton D. Ferns R. B. Raffle J. Grant P. Reidy M.. (2018). Use of whole-genome sequencing in the investigation of a nosocomial influenza virus outbreak. J. Infect. Dis. 218, 1485–1489. 10.1093/infdis/jiy335 29873767 PMC6151078

[B106] Hutspardol S. Essa M. Richardson S. Schechter T. Ali M. Krueger J.. (2015). Significant transplantation-related mortality from respiratory virus infections within the first one hundred days in children after hematopoietic stem cell transplantation. Biol. Blood Marrow Transplant. 21, 1802–1807. 10.1016/j.bbmt.2015.06.015 26117558 PMC7110880

[B107] Ison M. G. (2014). Optimum timing of oseltamivir: lessons from Bangladesh. Lancet Infect. Dis. 14, 88–89. 10.1016/s1473-3099(13)70287-1 24268592

[B108] Ison M. G. Hayden F. G. (2002). Viral infections in immunocompromised patients: what's new with respiratory viruses? Curr. Opin. Infect. Dis. 15, 355–367. 10.1097/00001432-200208000-00002 12130931

[B109] Issa N. C. Marty F. M. Gagne L. S. Koo S. Verrill K. A. Alyea E. P.. (2011). Seroprotective titers against 2009 H1N1 influenza A virus after vaccination in allogeneic hematopoietic stem cell transplantation recipients. Biol. Blood Marrow Transplant. 17, 434–438. 10.1016/j.bbmt.2010.10.002 20950701 PMC3262168

[B110] Jacobs S. E. Lamson D. M. St George K. Walsh T. J. (2013). Human rhinoviruses. Clin. Microbiol. Rev. 26, 135–162. 10.1128/cmr.00077-12 23297263 PMC3553670

[B111] Jorquera P. A. Tripp R. A. (2017). Respiratory syncytial virus: prospects for new and emerging therapeutics. Expert Rev. Respir. Med. 11, 609–615. 10.1080/17476348.2017.1338567 28574729

[B112] Kamboj M. Gerbin M. Huang C. K. Brennan C. Stiles J. Balashov S.. (2008). Clinical characterization of human metapneumovirus infection among patients with cancer. J. Infect. 57, 464–471. 10.1016/j.jinf.2008.10.003 19027169

[B113] Karron R. A. Wright P. F. Hall S. L. Makhene M. Thompson J. Burns B. A.. (1995a). A live attenuated bovine parainfluenza virus type 3 vaccine is safe, infectious, immunogenic, and phenotypically stable in infants and children. J. Infect. Dis. 171, 1107–1114. 7751684 10.1093/infdis/171.5.1107

[B114] Karron R. A. Wright P. F. Newman F. K. Makhene M. Thompson J. Samorodin R.. (1995b). A live human parainfluenza type 3 virus vaccine is attenuated and immunogenic in healthy infants and children. J. Infect. Dis. 172, 1445–1450. 7594701 10.1093/infdis/172.6.1445

[B115] Kassis C. Champlin R. E. Hachem R. Y. Hosing C. Tarrand J. J. Perego C. A.. (2010). Detection and control of a nosocomial respiratory syncytial virus outbreak in a stem cell transplantation unit: the role of palivizumab. Biol. Blood Marrow Transplant. 16, 1265–1271. 10.1016/j.bbmt.2010.03.011 20304082

[B116] Khanna N. Steffen I. Studt J. D. Schreiber A. Lehmann T. Weisser M.. (2009). Outcome of influenza infections in outpatients after allogeneic hematopoietic stem cell transplantation. Transpl. Infect. Dis. 11, 100–105. 10.1111/j.1399-3062.2008.00362.x 19175540

[B117] Kim Y. I. Pareek R. Murphy R. Harrison L. Farrell E. Cook R.. (2017). The antiviral effects of RSV fusion inhibitor, MDT-637, on clinical isolates, vs its achievable concentrations in the human respiratory tract and comparison to ribavirin. Influenza Other Respir. Viruses 11, 525–530. 10.1111/irv.12503 28990339 PMC5705693

[B118] Kim Y. J. Boeckh M. Englund J. A. (2007). Community respiratory virus infections in immunocompromised patients: hematopoietic stem cell and solid organ transplant recipients, and individuals with human immunodeficiency virus infection. Semin. Respir. Crit. Care Med. 28, 222–242. 10.1055/s-2007-976494 17458776

[B119] Kim Y. J. Guthrie K. A. Waghmare A. Walsh E. E. Falsey A. R. Kuypers J.. (2014). Respiratory syncytial virus in hematopoietic cell transplant recipients: factors determining progression to lower respiratory tract disease. J. Infect. Dis. 209, 1195–1204. 10.1093/infdis/jit832 24368837 PMC3969549

[B120] Kiso M. Takahashi K. Sakai-Tagawa Y. Shinya K. Sakabe S. Le Q. M.. (2010). T-705 (favipiravir) activity against lethal H5N1 influenza A viruses. Proc. Natl. Acad. Sci. U.S.A. 107, 882–887. 10.1073/pnas.0909603107 20080770 PMC2818889

[B121] Knight V. Gilbert B. E. Wyde P. R. Englund J. A. (1991). High dose-short duration ribavirin aerosol treatment–a review. Bull. Int. Union Tuberc. Lung Dis. 66, 97–101. 1756300

[B122] Koszalka P. Tilmanis D. Hurt A. C. (2017). Influenza antivirals currently in late-phase clinical trial. Influenza Other Respir. Viruses 11, 240–246. 10.1111/irv.12446 28146320 PMC5410715

[B123] Kothari A. Burgess M. J. Crescencio J. C. R. Kennedy J. L. Denson J. L. Schwalm K. C.. (2017). The role of next generation sequencing in infection prevention in human parainfluenza virus 3 infections in immunocompromised patients. J. Clin. Virol. 92, 53–55. 10.1016/j.jcv.2017.05.010 28531552 PMC5521260

[B124] Kou M. Hwang V. Ramkellawan N. (2018). Bronchiolitis: From practice guideline to clinical practice. Emerg. Med. Clin. North Am. 36, 275–286. 10.1016/j.emc.2017.12.006 29622322

[B125] Kumar D. Ferreira V. H. Blumberg E. Silveira F. Cordero E. Perez-Romero P. (2018). A five-year prospective multi-center evaluation of influenza infection in transplant recipients. Clin. Infect. Dis. 67, 1322–1329. 10.1093/cid/ciy294 29635437

[B126] Laplante J. St George K. (2014). Antiviral resistance in influenza viruses: laboratory testing. Clin. Lab. Med. 34, 387–408. 10.1016/j.cll.2014.02.010 24856534

[B127] Lee N. Chan P. K. Wong C. K. Wong K. T. Choi K. W. Joynt G. M.. (2011). Viral clearance and inflammatory response patterns in adults hospitalized for pandemic 2009 influenza A(H1N1) virus pneumonia. Antivir. Ther. 16, 237–247. 10.3851/imp1722 21447873

[B128] Leen A. M. Heslop H. E. Brenner M. K. (2014). Antiviral T-cell therapy. Immunol. Rev. 258, 12–29. 10.1111/imr.12138 24517423 PMC3927231

[B129] Ljungman P. (2012). Vaccination of immunocompromised patients. Clin. Microbiol. Infect. 18(Suppl. 5), 93–99. 10.1111/j.1469-0691.2012.03971.x 23051059

[B130] Ljungman P. de la Camara R. Perez-Bercoff L. Abecasis M. Nieto Campuzano J. B. Cannata-Ortiz M. J.. (2011). Outcome of pandemic H1N1 infections in hematopoietic stem cell transplant recipients. Haematologica 96, 1231–1235. 10.3324/haematol.2011.041913 21546495 PMC3148919

[B131] Ljungman P. Ward K. N. Crooks B. N. Parker A. Martino R. Shaw P. J.. (2001). Respiratory virus infections after stem cell transplantation: a prospective study from the Infectious Diseases Working Party of the European Group for Blood and Marrow Transplantation. Bone Marrow Transplant. 28, 479–484. 10.1038/sj.bmt.1703139 11593321

[B132] Loria C. Domm J. A. Halasa N. B. Heitman E. Miller E. K. Xu M.. (2015). Human rhinovirus C infections in pediatric hematology and oncology patients. Pediatr. Transplant. 19, 94–100. 10.1111/petr.12383 25377237 PMC4280346

[B133] Louie J. K. Yang S. Acosta M. Yen C. Samuel M. C. Schechter R.. (2012a). Treatment with neuraminidase inhibitors for critically ill patients with influenza A (H1N1)pdm09. Clin. Infect. Dis. 55, 1198–1204. 10.1093/cid/cis636 22843781 PMC12362346

[B134] Louie J. K. Yang S. Yen C. Acosta M. Schechter R. Uyeki T. M. (2012b). Use of intravenous peramivir for treatment of severe influenza A(H1N1)pdm09. PLoS ONE 7:e40261. 10.1371/journal.pone.0040261 22768265 PMC3386960

[B135] Luján-Zilbermann J. Benaim E. Tong X. Srivastava D. K. Patrick C. C. DeVincenzo J. P. (2001). Respiratory virus infections in pediatric hematopoietic stem cell transplantation. Clin. Infect. Dis. 33, 962–968. 10.1086/322628 11528566

[B136] Lukens M. V. van de Pol A. C. Coenjaerts F. E. Jansen N. J. Kamp V. M. Kimpen J. L.. (2010). A systemic neutrophil response precedes robust CD8(+) T-cell activation during natural respiratory syncytial virus infection in infants. J. Virol. 84, 2374–2383. 10.1128/jvi.01807-09 20015982 PMC2820924

[B137] Machado C. M. Cardoso M. R. da Rocha I. F. Boas L. S. Dulley F. L. Pannuti C. S. (2005). The benefit of influenza vaccination after bone marrow transplantation. Bone Marrow Transplant. 36, 897–900. 10.1038/sj.bmt.1705159 16170332

[B138] Maeng S. H. Yoo H. S. Choi S. H. Yoo K. H. Kim Y. J. Sung K. W.. (2012). Impact of parainfluenza virus infection in pediatric cancer patients. Pediatr. Blood Cancer 59, 708–710. 10.1002/pbc.23390 22095941

[B139] Mahony J. B. (2008). Detection of respiratory viruses by molecular methods. Clin. Microbiol. Rev. 21, 716–747. 10.1128/cmr.00037-07 18854489 PMC2570148

[B140] Mahony J. B. Petrich A. Smieja M. (2011). Molecular diagnosis of respiratory virus infections. Crit. Rev. Clin. Lab. Sci. 48, 217–249. 10.3109/10408363.2011.640976 22185616

[B141] Marjuki H. Mishin V. P. Sleeman K. Okomo-Adhiambo M. Sheu T. G. Guo L.. (2013). Bioluminescence-based neuraminidase inhibition assay for monitoring influenza virus drug susceptibility in clinical specimens. Antimicrob. Agents Chemother. 57, 5209–5215. 10.1128/aac.01086-13 23917311 PMC3811246

[B142] Martino R. Porras R. P. Rabella N. Williams J. V. Ramila E. Margall N.. (2005). Prospective study of the incidence, clinical features, and outcome of symptomatic upper and lower respiratory tract infections by respiratory viruses in adult recipients of hematopoietic stem cell transplants for hematologic malignancies. Biol. Blood Marrow Transplant. 11, 781–796. 10.1016/j.bbmt.2005.07.007 16182179 PMC3347977

[B143] Matz J. (2013). Vapendavir significantly improves upper respiratory symptoms of naturally acquired rhinovirus infection in asthmatic adults: Results of a phase 2 clinical trial. Eur. Respir. J. 42(Suppl 57), 1493.

[B144] McLellan J. S. Ray W. C. Peeples M. E. (2013). Structure and function of respiratory syncytial virus surface glycoproteins. Curr. Top. Microbiol. Immunol. 372, 83–104. 10.1007/978-3-642-38919-1_4 24362685 PMC4211642

[B145] Meissner H. C. (2016). Viral bronchiolitis in children. N. Engl. J. Med. 374, 62–72. 10.1056/NEJMra1413456 26735994

[B146] Mejias A. Garcia-Maurino C. Rodriguez-Fernandez R. Peeples M. E. Ramilo O. (2017). Development and clinical applications of novel antibodies for prevention and treatment of respiratory syncytial virus infection. Vaccine 35, 496–502. 10.1016/j.vaccine.2016.09.026 27692523 PMC5183505

[B147] Melero J. A. Mas V. McLellan J. S. (2017). Structural, antigenic and immunogenic features of respiratory syncytial virus glycoproteins relevant for vaccine development. Vaccine 35, 461–468. 10.1016/j.vaccine.2016.09.045 27692522 PMC5189713

[B148] Milano F. Campbell A. P. Guthrie K. A. Kuypers J. Englund J. A. Corey L.. (2010). Human rhinovirus and coronavirus detection among allogeneic hematopoietic stem cell transplantation recipients. Blood 115, 2088–2094. 10.1182/blood-2009-09-244152 20042728 PMC2837322

[B149] Moss R. B. Davey R. T. Steigbigel R. T. Fang F. (2010). Targeting pandemic influenza: a primer on influenza antivirals and drug resistance. J. Antimicrob. Chemother. 65, 1086–1093. 10.1093/jac/dkq100 20375034

[B150] Moss R. B. Hansen C. Sanders R. L. Hawley S. Li T. Steigbigel R. T. (2012). A phase II study of DAS181, a novel host directed antiviral for the treatment of influenza infection. J. Infect. Dis. 206, 1844–1851. 10.1093/infdis/jis622 23045618 PMC3570175

[B151] Mulrennan S. Tempone S. S. Ling I. T. Williams S. H. Gan G. C. Murray R. J.. (2010). Pandemic influenza (H1N1) 2009 pneumonia: CURB-65 score for predicting severity and nasopharyngeal sampling for diagnosis are unreliable. PLoS ONE 5:e12849. 10.1371/journal.pone.0012849 20877727 PMC2943473

[B152] Nichols W. G. Corey L. Gooley T. Davis C. Boeckh M. (2001). Parainfluenza virus infections after hematopoietic stem cell transplantation: risk factors, response to antiviral therapy, and effect on transplant outcome. Blood 98, 573–578. 10.1182/blood.v98.3.573 11468152

[B153] Nichols W. G. Guthrie K. A. Corey L. Boeckh M. (2004). Influenza infections after hematopoietic stem cell transplantation: risk factors, mortality, and the effect of antiviral therapy. Clin. Infect. Dis. 39, 1300–1306. 10.1086/425004 15494906

[B154] Nicholson K. G. Wood J. M. Zambon M. (2003). Influenza. Lancet 362, 1733–1745. 10.1016/s0140-6736(03)14854-4 14643124 PMC7112395

[B155] Ogimi C. Englund J. A. Bradford M. C. Qin X. Boeckh M. Waghmare A. (2018a). Characteristics and outcomes of coronavirus infection in children: the role of viral factors and an immunocompromised state. J. Pediatric Infect. Dis. Soc. pix093. 10.1093/jpids/pix093 29447395 PMC6437838

[B156] Ogimi C. Greninger A. L. Waghmare A. A. Kuypers J. M. Shean R. C. Xie H.. (2017a). Prolonged shedding of human coronavirus in hematopoietic cell transplant recipients: risk factors and viral genome evolution. J. Infect. Dis. 216, 203–209. 10.1093/infdis/jix264 28838146 PMC5853311

[B157] Ogimi C. Waghmare A. A. Kuypers J. M. Xie H. Yeung C. C. Leisenring W. M.. (2017b). Clinical significance of human coronavirus in bronchoalveolar lavage samples from hematopoietic cell transplant recipients and patients with hematologic malignancies. Clin. Infect. Dis. 64, 1532–1539. 10.1093/cid/cix160 28329354 PMC5434339

[B158] Ogimi C. Xie H. Leisenring W. M. Kuypers J. M. Jerome K. R. Campbell A. P.. (2018b). Initial high viral load is associated with prolonged shedding of human rhinovirus in allogeneic hematopoietic cell transplant recipients. Biol. Blood Marrow Transplant. 24, 2160–2163. 10.1016/j.bbmt.2018.07.006 30009982 PMC6239940

[B159] Okomo-Adhiambo M. Fry A. M. Su S. Nguyen H. T. Elal A. A. Negron E.. (2015). Oseltamivir-resistant influenza A(H1N1)pdm09 viruses, United States, 2013-14. Emerging Infect. Dis. 21, 136–141. 10.3201/eid2101.141006 25532050 PMC4285251

[B160] Okomo-Adhiambo M. Nguyen H. T. Abd Elal A. Sleeman K. Fry A. M. Gubareva L. V. (2014). Drug susceptibility surveillance of influenza viruses circulating in the United States in 2011-2012: application of the WHO antiviral working group criteria. Influenza Other Respir. Viruses 8, 258–265. 10.1111/irv.12215 24299049 PMC4186475

[B161] Okomo-Adhiambo M. Sheu T. G. Gubareva L. V. (2013). Assays for monitoring susceptibility of influenza viruses to neuraminidase inhibitors. Influenza Other Respir. Viruses 7(Suppl. 1), 44–49. 10.1111/irv.12051 23279896 PMC5978623

[B162] Omoto S. Speranzini V. Hashimoto T. Noshi T. Yamaguchi H. Kawai M.. (2018). Characterization of influenza virus variants induced by treatment with the endonuclease inhibitor baloxavir marboxil. Sci. Rep. 8, 9633. 10.1038/s41598-018-27890-4 29941893 PMC6018108

[B163] Ottolini M. G. Porter D. D. Hemming V. G. Zimmerman M. N. Schwab N. M. Prince G. A. (1999). Effectiveness of RSVIG prophylaxis and therapy of respiratory syncytial virus in an immunosuppressed animal model. Bone Marrow Transplant. 24, 41–45. 10.1038/sj.bmt.1701813 10435733

[B164] Papenburg J. Boivin G. (2010). The distinguishing features of human metapneumovirus and respiratory syncytial virus. Rev. Med. Virol. 20, 245–260. 10.1002/rmv.651 20586081

[B165] Pasikhova Y. Hayne J. Baluch A. (2018). Oral Ribavirin for the Treatment of Respiratory Syncytial Virus (RSV) and Human Metapneumovirus (hMPV) Infections in Hematology Patients and Stem Cell Transplant (SCT) Recipients at a Nci-Designated Cancer Center. Biol. Blood Marrow Transplant. 24:S383 10.1016/j.bbmt.2017.12.471

[B166] Peck A. J. Englund J. A. Kuypers J. Guthrie K. A. Corey L. Morrow R.. (2007). Respiratory virus infection among hematopoietic cell transplant recipients: evidence for asymptomatic parainfluenza virus infection. Blood 110, 1681–1688. 10.1182/blood-2006-12-060343 17502457 PMC1975849

[B167] Popowitch E. B. O'Neill S. S. Miller M. B. (2013). Comparison of the Biofire FilmArray RP, Genmark eSensor RVP, Luminex xTAG RVPv1, and Luminex xTAG RVP fast multiplex assays for detection of respiratory viruses. J. Clin. Microbiol. 51, 1528–1533. 10.1128/jcm.03368-12 23486707 PMC3647947

[B168] Pyrc K. Berkhout B. van der Hoek L. (2007). Antiviral strategies against human coronaviruses. Infect. Disord. Drug Targets 7, 59–66. 10.2174/187152607780090757 17346212

[B169] Raad I. Abbas J. Whimbey E. (1997). Infection control of nosocomial respiratory viral disease in the immunocompromised host. Am. J. Med. 102, 48–52*;* discussion 53–44. 10868143 10.1016/s0002-9343(97)00011-9

[B170] Rand K. H. Rampersaud H. Houck H. J. (2011). Comparison of two multiplex methods for detection of respiratory viruses: filmArray RP and xTAG RVP. J. Clin. Microbiol. 49, 2449–2453. 10.1128/jcm.02582-10 21508156 PMC3147849

[B171] Renaud C. Boudreault A. A. Kuypers J. Lofy K. H. Corey L. Boeckh M. J.. (2011a). H275Y mutant pandemic (H1N1) 2009 virus in immunocompromised patients. Emerg. Infect. Dis. 17, 653–660; quiz 765. 10.3201/eid1704.101429 21470455 PMC3290123

[B172] Renaud C. Campbell A. P. (2011). Changing epidemiology of respiratory viral infections in hematopoietic cell transplant recipients and solid organ transplant recipients. Curr. Opin. Infect. Dis. 24, 333–343. 10.1097/QCO.0b013e3283480440 21666460 PMC3210111

[B173] Renaud C. Englund J. A. (2012). Antiviral therapy of respiratory viruses in haematopoietic stem cell transplant recipients. Antivir. Ther. 17, 175–191. 10.3851/imp2060 22311587

[B174] Renaud C. Kuypers J. Aspesberro F. Rosenfeld M. Englund J. A. (2011c). Emergence of oseltamivir-resistant pandemic H1N1 in an immunocompetent child with severe status asthmaticus. J. Asthma 48, 572–575. 10.3109/02770903.2011.582660 21604924

[B175] Renaud C. Kuypers J. Englund J. A. (2011b). Emerging oseltamivir resistance in seasonal and pandemic influenza A/H1N1. J. Clin. Virol. 52, 70–78. 10.1016/j.jcv.2011.05.019 21684202

[B176] Renaud C. Pergam S. A. Polyak C. Jain R. Kuypers J. Englund J. A.. (2010). Early emergence of an H275Y mutation in a hematopoietic cell transplant recipient treated with intravenous peramivir. Transpl. Infect. Dis. 12, 513–517. 10.1111/j.1399-3062.2010.00582.x 21062390 PMC3024056

[B177] Renaud C. Xie H. Seo S. Kuypers J. Cent A. Corey L.. (2013). Mortality rates of human metapneumovirus and respiratory syncytial virus lower respiratory tract infections in hematopoietic cell transplantation recipients. Biol. Blood Marrow Transplant. 19, 1220–1226. 10.1016/j.bbmt.2013.05.005 23680472 PMC3752411

[B178] Rossey I. McLellan J. S. Saelens X. Schepens B. (2018). Clinical potential of prefusion RSV F-specific antibodies. Trends Microbiol. 26, 209–219. 10.1016/j.tim.2017.09.009 29054341

[B179] Rossignol J. F. (2014). Nitazoxanide: a first-in-class broad-spectrum antiviral agent. Antiviral Res. 110, 94–103. 10.1016/j.antiviral.2014.07.014 25108173 PMC7113776

[B180] Roymans D. Alnajjar S. S. Battles M. B. Sitthicharoenchai P. Furmanova-Hollenstein P. Rigaux P.. (2017). Therapeutic efficacy of a respiratory syncytial virus fusion inhibitor. Nat. Commun. 8:167. 10.1038/s41467-017-00170-x 28761099 PMC5537225

[B181] Rubin L. G. Levin M. J. Ljungman P. Davies E. G. Avery R. Tomblyn M. (2014). 2013 IDSA clinical practice guideline for vaccination of the immunocompromised host. Clin. Infect. Dis. 58, 309–318. 10.1093/cid/cit816 24421306

[B182] Salez N. Vabret A. Leruez-Ville M. Andreoletti L. Carrat F. Renois F.. (2015). Evaluation of four commercial multiplex molecular tests for the diagnosis of acute respiratory infections. PLoS ONE 10:e0130378. 10.1371/journal.pone.0130378 26107509 PMC4481272

[B183] Samuel S. Nanjappa S. Cooper C. D. Greene J. N. (2016). Human metapneumovirus infection in immunocompromised patients. Cancer Control 23, 442–445. 10.1177/107327481602300416 27842334

[B184] Schildgen V. van den Hoogen B. Fouchier R. Tripp R. A. Alvarez R. Manoha C.. (2011). Human metapneumovirus: lessons learned over the first decade. Clin. Microbiol. Rev. 24, 734–754. 10.1128/cmr.00015-11 21976607 PMC3194831

[B185] Seo S. Campbell A. P. Xie H. Chien J. W. Leisenring W. M. Englund J. A.. (2013). Outcome of respiratory syncytial virus lower respiratory tract disease in hematopoietic cell transplant recipients receiving aerosolized ribavirin: significance of stem cell source and oxygen requirement. Biol. Blood Marrow Transplant. 19, 589–596. 10.1016/j.bbmt.2012.12.019 23298855 PMC3667608

[B186] Seo S. Gooley T. A. Kuypers J. M. Stednick Z. Jerome K. R. Englund J. A.. (2016). Human metapneumovirus infections following hematopoietic cell transplantation: factors associated with disease progression. Clin. Infect. Dis. 63, 178–185. 10.1093/cid/ciw284 27143659 PMC4928387

[B187] Seo S. Waghmare A. Scott E. M. Xie H. Kuypers J. M. Hackman R. C.. (2017). Human rhinovirus detection in the lower respiratory tract of hematopoietic cell transplant recipients: association with mortality. Haematologica 102, 1120–1130. 10.3324/haematol.2016.153767 28183847 PMC5451345

[B188] Seo S. Xie H. Campbell A. P. Kuypers J. M. Leisenring W. M. Englund J. A.. (2014). Parainfluenza virus lower respiratory tract disease after hematopoietic cell transplant: viral detection in the lung predicts outcome. Clin. Infect. Dis. 58, 1357–1368. 10.1093/cid/ciu134 24599766 PMC4001290

[B189] Shah D. P. Ghantoji S. S. Ariza-Heredia E. J. Shah J. N. El Taoum K. K. Shah P. K.. (2014). Immunodeficiency scoring index to predict poor outcomes in hematopoietic cell transplant recipients with RSV infections. Blood 123, 3263–3268. 10.1182/blood-2013-12-541359 24700783 PMC4046424

[B190] Shah D. P. Ghantoji S. S. Azzi J. Avadhanula V. Kmeid J. El Chaer F. (2016a). An open label, block-randomized, ribavirin efficacy trial for management of RSV infections in Hematopoietic Cell Transplant (HCT) recipients: clinical and economic implications. Biol. Blood Marrow Transplant. 22, S52–S53. 10.1016/j.bbmt.2015.11.338

[B191] Shah D. P. Ghantoji S. S. Mulanovich V. E. Ariza-heredia E. J. Chemaly R. F. (2012). Management of respiratory viral infections in hematopoietic cell transplant recipients. Am. J. Blood Res. 2, 203–218. 23226621 PMC3512176

[B192] Shah D. P. Shah P. K. Azzi J. M. Chemaly R. F. (2016b). Parainfluenza virus infections in hematopoietic cell transplant recipients and hematologic malignancy patients: a systematic review. Cancer Lett. 370, 358–364. 10.1016/j.canlet.2015.11.014 26582658 PMC4684719

[B193] Shah D. P. Shah P. K. Azzi J. M. El Chaer F. Chemaly R. F. (2016c). Human metapneumovirus infections in hematopoietic cell transplant recipients and hematologic malignancy patients: a systematic review. Cancer Lett. 379, 100–106. 10.1016/j.canlet.2016.05.035 27260872 PMC4935561

[B194] Shah J. N. Chemaly R. F. (2011). Management of RSV infections in adult recipients of hematopoietic stem cell transplantation. Blood 117, 2755–2763. 10.1182/blood-2010-08-263400 21139081

[B195] Shahani L. Ariza-Heredia E. J. Chemaly R. F. (2017). Antiviral therapy for respiratory viral infections in immunocompromised patients. Expert Rev. Anti Infect. Ther. 15, 401–415. 10.1080/14787210.2017.1279970 28067078 PMC7103713

[B196] Sleeman K. Mishin V. P. Deyde V. M. Furuta Y. Klimov A. I. Gubareva L. V. (2010). In vitro antiviral activity of favipiravir (T-705) against drug-resistant influenza and 2009 A(H1N1) viruses. Antimicrob. Agents Chemother. 54, 2517–2524. 10.1128/aac.01739-09 20350949 PMC2876376

[B197] Srinivasan A. Wang C. Yang J. Inaba H. Shenep J. L. Leung W. H.. (2011a). Parainfluenza virus infections in children with hematologic malignancies. Pediatr. Infect. Dis. J. 30, 855–859. 10.1097/INF.0b013e31821d190f 21540759 PMC3196524

[B198] Srinivasan A. Wang C. Yang J. Shenep J. L. Leung W. H. Hayden R. T. (2011b). Symptomatic parainfluenza virus infections in children undergoing hematopoietic stem cell transplantation. Biol. Blood Marrow Transplant. 17, 1520–1527. 10.1016/j.bbmt.2011.03.001 21396476 PMC4936785

[B199] Stevens M. Rusch S. DeVincenzo J. Kim Y. I. Harrison L. Meals E. A.. (2018). Antiviral activity of oral JNJ-53718678 in healthy adult volunteers challenged with respiratory syncytial virus: a placebo-controlled study. J. Infect. Dis. 218, 748–756. 10.1093/infdis/jiy227 29684148

[B200] Sykes A. Gerhardt E. Tang L. Adderson E. E. (2017). The effectiveness of trivalent inactivated influenza vaccine in children with acute leukemia. J. Pediatr. 191, 218–224.e211. 10.1016/j.jpeds.2017.08.071 29173310 PMC5726795

[B201] Tamura D. Okomo-Adhiambo M. Mishin V. P. Guo Z. Xu X. Villanueva J.. (2015). Application of a seven-target pyrosequencing assay to improve the detection of neuraminidase inhibitor-resistant Influenza A(H3N2) viruses. Antimicrob. Agents Chemother. 59, 2374–2379. 10.1128/aac.04939-14 25645846 PMC4356763

[B202] Tantawy A. A. Barakat M. M. Adly A. A. Ebeid F. S. Shamaa M. F. Yassin M. (2015). One-year prospective study of community acquired influenza and parainfluenza viral infections in hospitalized egyptian children with malignancy: single center experience. Pediatr. Hematol. Oncol. 32, 304–314. 10.3109/08880018.2015.1013230 25871509

[B203] Tian D. Battles M. B. Moin S. M. Chen M. Modjarrad K. Kumar A.. (2017). Structural basis of respiratory syncytial virus subtype-dependent neutralization by an antibody targeting the fusion glycoprotein. Nat. Commun. 8, 1877. 10.1038/s41467-017-01858-w 29187732 PMC5707411

[B204] Tilmanis D. van Baalen C. Oh D. Y. Rossignol J. F. Hurt A. C. (2017). The susceptibility of circulating human influenza viruses to tizoxanide, the active metabolite of nitazoxanide. Antiviral Res. 147, 142–148. 10.1016/j.antiviral.2017.10.002 28986103

[B205] Tomblyn M. Chiller T. Einsele H. Gress R. Sepkowitz K. Storek J.. (2009). Guidelines for preventing infectious complications among hematopoietic cell transplantation recipients: a global perspective. Biol. Blood Marrow Transplant. 15, 1143–1238. 10.1016/j.bbmt.2009.06.019 19747629 PMC3103296

[B206] Tregoning J. S. Schwarze J. (2010). Respiratory viral infections in infants: causes, clinical symptoms, virology, and immunology. Clin. Microbiol. Rev. 23, 74–98. 10.1128/cmr.00032-09 20065326 PMC2806659

[B207] Triana-Baltzer G. B. Gubareva L. V. Klimov A. I. Wurtman D. F. Moss R. B. Hedlund M.. (2009a). Inhibition of neuraminidase inhibitor-resistant influenza virus by DAS181, a novel sialidase fusion protein. PLoS ONE 4:e7838. 10.1371/journal.pone.0007838 19893749 PMC2770896

[B208] Triana-Baltzer G. B. Gubareva L. V. Nicholls J. M. Pearce M. B. Mishin V. P. Belser J. A.. (2009b). Novel pandemic influenza A(H1N1) viruses are potently inhibited by DAS181, a sialidase fusion protein. PLoS ONE 4:e7788. 10.1371/journal.pone.0007788 19893747 PMC2770640

[B209] Tzannou I. Nicholas S. K. Lulla P. Aguayo-Hiraldo P. I. Misra A. Martinez C. A.. (2017). Immunologic profiling of human metapneumovirus for the development of targeted immunotherapy. J. Infect. Dis. 216, 678–687. 10.1093/infdis/jix358 28934427 PMC5853664

[B210] Ulbrandt N. D. Ji H. Patel N. K. Riggs J. M. Brewah Y. A. Ready S.. (2006). Isolation and characterization of monoclonal antibodies which neutralize human metapneumovirus *in vitro* and *in vivo*. J. Virol. 80, 7799–7806. 10.1128/jvi.00318-06 16873237 PMC1563801

[B211] Ustun C. Slaby J. Shanley R. M. Vydra J. Smith A. R. Wagner J. E.. (2012). Human parainfluenza virus infection after hematopoietic stem cell transplantation: risk factors, management, mortality, and changes over time. Biol. Blood Marrow Transplant. 18, 1580–1588. 10.1016/j.bbmt.2012.04.012 22531491 PMC3443286

[B212] van der Hoek L. (2007). Human coronaviruses: what do they cause? Antivir. Ther. 12(4 Pt B), 651–658. 17944272

[B213] Vilchez R. A. Dauber J. McCurry K. Iacono A. Kusne S. (2003). Parainfluenza virus infection in adult lung transplant recipients: an emergent clinical syndrome with implications on allograft function. Am. J. Transplant 3, 116–120. 10.1034/j.1600-6143.2003.00024.x 12603206

[B214] Vilchez R. A. McCurry K. Dauber J. Iacono A. Keenan R. Zeevi A.. (2001). The epidemiology of parainfluenza virus infection in lung transplant recipients. Clin. Infect. Dis. 33, 2004–2008. 10.1086/324348 11702289

[B215] Waghmare A. Campbell A. P. Xie H. Seo S. Kuypers J. Leisenring W.. (2013). Respiratory syncytial virus lower respiratory disease in hematopoietic cell transplant recipients: viral RNA detection in blood, antiviral treatment, and clinical outcomes. Clin. Infect. Dis. 57, 1731–1741. 10.1093/cid/cit639 24065324 PMC3840404

[B216] Waghmare A. Englund J. A. Boeckh M. (2016). How I treat respiratory viral infections in the setting of intensive chemotherapy or hematopoietic cell transplantation. Blood 127, 2682–2692. 10.1182/blood-2016-01-634873 26968533 PMC4891952

[B217] Waghmare A. Wagner T. Andrews R. Smith S. Kuypers J. Boeckh M.. (2015). Successful treatment of parainfluenza virus respiratory tract infection with DAS181 in 4 immunocompromised children. J. Pediatric Infect. Dis. Soc. 4, 114–118. 10.1093/jpids/piu039 26185620 PMC4501511

[B218] Walsh E. E. McConnochie K. M. Long C. E. Hall C. B. (1997). Severity of respiratory syncytial virus infection is related to virus strain. J. Infect. Dis. 175, 814–820. 9086135 10.1086/513976

[B219] Walsh E. E. Wang L. Falsey A. R. Qiu X. Corbett A. Holden-Wiltse J.. (2018). Virus-specific antibody, viral load, and disease severity in respiratory syncytial virus infection. J. Infect. Dis. 218, 208–217. 10.1093/infdis/jiy106 29546402 PMC6009588

[B220] Watanabe A. Chang S. C. Kim M. J. Chu D. W. Ohashi Y. (2010). Long-acting neuraminidase inhibitor laninamivir octanoate versus oseltamivir for treatment of influenza: a double-blind, randomized, noninferiority clinical trial. Clin. Infect. Dis. 51, 1167–1175. 10.1086/656802 20936975

[B221] Watcharananan S. P. Suwatanapongched T. Wacharawanichkul P. Chantratitaya W. Mavichak V. Mossad S. B. (2010). Influenza A/H1N1 2009 pneumonia in kidney transplant recipients: characteristics and outcomes following high-dose oseltamivir exposure. Transpl. Infect. Dis. 12, 127–131. 10.1111/j.1399-3062.2010.00493.x 20102550

[B222] Weigt S. S. Gregson A. L. Deng J. C. Lynch J. P. 3rd Belperio J. A. (2011). Respiratory viral infections in hematopoietic stem cell and solid organ transplant recipients. Semin. Respir. Crit. Care Med. 32, 471–493. 10.1055/s-0031-1283286 21858751 PMC4209842

[B223] Whimbey E. Champlin R. E. Couch R. B. Englund J. A. Goodrich J. M. Raad I.. (1996). Community respiratory virus infections among hospitalized adult bone marrow transplant recipients. Clin. Infect. Dis. 22, 778–782. 8722930 10.1093/clinids/22.5.778

[B224] Whimbey E. Champlin R. E. Englund J. A. Mirza N. Q. Piedra P. A. Goodrich J. M.. (1995). Combination therapy with aerosolized ribavirin and intravenous immunoglobulin for respiratory syncytial virus disease in adult bone marrow transplant recipients. Bone Marrow Transplant. 16, 393–399. 8535312

[B225] Whimbey E. Englund J. A. Couch R. B. (1997). Community respiratory virus infections in immunocompromised patients with cancer. Am. J. Med. 102, 10–18; discussion 25–16. 10868137 10.1016/S0002-9343(97)80004-6 PMC7172994

[B226] Yeh E. Luo R. F. Dyner L. Hong D. K. Banaei N. Baron E. J.. (2010). Preferential lower respiratory tract infection in swine-origin 2009 A(H1N1) influenza. Clin. Infect. Dis. 50, 391–394. 10.1086/649875 20047483

[B227] Zhu Q. McLellan J. S. Kallewaard N. L. Ulbrandt N. D. Palaszynski S. Zhang J.. (2017). A highly potent extended half-life antibody as a potential RSV vaccine surrogate for all infants. Sci. Transl. Med. 9:eaaj1928. 10.1126/scitranslmed.aaj1928 28469033

[B228] Zhu Y. Zembower T. R. Metzger K. E. Lei Z. Green S. J. Qi C. (2017). Investigation of respiratory syncytial virus outbreak on an adult stem cell transplant unit by use of whole-genome sequencing. J. Clin. Microbiol. 55, 2956–2963. 10.1128/jcm.00360-17 28747373 PMC5625381

